# Pacific Biosciences Sequencing and IMGT/HighV-QUEST Analysis of Full-Length Single Chain Fragment Variable from an *In Vivo* Selected Phage-Display Combinatorial Library

**DOI:** 10.3389/fimmu.2017.01796

**Published:** 2017-12-20

**Authors:** Audrey Hemadou, Véronique Giudicelli, Melissa Laird Smith, Marie-Paule Lefranc, Patrice Duroux, Sofia Kossida, Cheryl Heiner, N. Lance Hepler, John Kuijpers, Alexis Groppi, Jonas Korlach, Philippe Mondon, Florence Ottones, Marie-Josée Jacobin-Valat, Jeanny Laroche-Traineau, Gisèle Clofent-Sanchez

**Affiliations:** ^1^CRMSB, UMR 5536, CNRS, Bordeaux, France; ^2^IMGT^®^, The International ImMunoGeneTics Information System^®^, Laboratoire d’ImmunoGénétique Moléculaire, LIGM, Institut de Génétique Humaine, IGH, UMR 9002, CNRS, Montpellier University, Montpellier, France; ^3^Pacific Biosciences, Menlo Park, CA, United States; ^4^Université de Bordeaux, Centre de Bioinformatique de Bordeaux (CBiB), Bordeaux, France; ^5^LFB Biotechnologies, Lille, France

**Keywords:** human antibody, IMGT/HighV-QUEST, immunoinformatics, immunoglobulin, Pacific Biosciences sequencing, phage combinatorial library, single chain fragment variable, next-generation sequencing

## Abstract

Phage-display selection of immunoglobulin (IG) or antibody single chain Fragment variable (scFv) from combinatorial libraries is widely used for identifying new antibodies for novel targets. Next-generation sequencing (NGS) has recently emerged as a new method for the high throughput characterization of IG and T cell receptor (TR) immune repertoires both *in vivo* and *in vitro*. However, challenges remain for the NGS sequencing of scFv from combinatorial libraries owing to the scFv length (>800 bp) and the presence of two variable domains [variable heavy (VH) and variable light (VL) for IG] associated by a peptide linker in a single chain. Here, we show that single-molecule real-time (SMRT) sequencing with the Pacific Biosciences RS II platform allows for the generation of full-length scFv reads obtained from an *in vivo* selection of scFv-phages in an animal model of atherosclerosis. We first amplified the DNA of the phagemid inserts from scFv-phages eluted from an aortic section at the third round of the *in vivo* selection. From this amplified DNA, 450,558 reads were obtained from 15 SMRT cells. Highly accurate circular consensus sequences from these reads were generated, filtered by quality and then analyzed by IMGT/HighV-QUEST with the functionality for scFv. Full-length scFv were identified and characterized in 348,659 reads. Full-length scFv sequencing is an absolute requirement for analyzing the associated VH and VL domains enriched during the *in vivo* panning rounds. In order to further validate the ability of SMRT sequencing to provide high quality, full-length scFv sequences, we tracked the reads of an scFv-phage clone P3 previously identified by biological assays and Sanger sequencing. Sixty P3 reads showed 100% identity with the full-length scFv of 767 bp, 53 of them covering the whole insert of 977 bp, which encompassed the primer sequences. The remaining seven reads were identical over a shortened length of 939 bp that excludes the vicinity of primers at both ends. Interestingly these reads were obtained from each of the 15 SMRT cells. Thus, the SMRT sequencing method and the IMGT/HighV-QUEST functionality for scFv provides a straightforward protocol for characterization of full-length scFv from combinatorial phage libraries.

## Introduction

Immunoglobulin (IG) or antibody fragments displayed as single chain Fragment variable (scFv) on filamentous phages (scFv-phages) are classically selected from scFv-phage combinatorial libraries to obtain human antibodies specific for a given target (1–3). This selection from scFv-phage display libraries is widely used for the discovery of novel specificities for therapeutic antibodies in cancer, cardiovascular, autoimmune, infectious or neurodegenerative pathologies, with many of them at various stages of clinical or research development (4–10). Classical *in vitro* phage display approaches involve multiple rounds of selection (or panning) for the enrichment of scFv-phages that demonstrate the desired specificity against a target followed, at the last selection round, by functional screening and characterization of selected candidates using appropriate assays. At this very last step, analysis of the selected scFv *via* Sanger sequencing is commonly used to identify sequences of interest, taking advantage of the genotype–phenotype linkage inherent to the display system. A critical limitation of using biological assays followed by Sanger sequencing is that only a minute fraction of the selected library is actually sampled, a few hundred at best, whereas the selected library may usually contain up to 10^5^ to 10^6^ variants. This limitation is further enhanced when scFv-phage selection is performed *in vivo* (biopanning) in different pathological models in which scFv-phages can encounter a very large panel of unknown biomarkers (11–13). Currently available next-generation sequencing (NGS) platforms allow the simultaneous sequencing of millions of reads. However, a main challenge for the NGS sequencing of scFv from combinatorial libraries remains the scFv length >800 bp, which is too long for most NGS platforms. Up to now, NGS methods have only provided reads encompassing one variable (V) domain (400 bp), therefore losing a critical piece of information found in scFv sequences, that of the association of two specific V domains [variable heavy (VH) and variable light (VL) for the IG] by the peptide linker. Although a few approaches have been proposed, retrieving information regarding V domain association has still not been solved (14–16).

The analysis of antibody scFv sequences is a difficult exercise because not only are scFv composed of two V domains, but these two V domains are different from each other and each can potentially be extremely diverse. Indeed, the huge diversity of IG or antibodies results from complex *in vivo* mechanisms that occur during the synthesis of the VH and VL domains, which include the molecular rearrangements at the DNA level of the variable (V), diversity (D) (only for VH), and joining (J) genes with nucleotide deletions and insertions (N-diversity) at the V-(D)-J junctions in the bone marrow pre-B and immature B cells (17, 18). In spleen and lymph nodes, somatic hypermutations accumulate in the mature B cell VH and VL, creating a huge diversity of the B cell membrane IG for the recognition of foreign antigens. Following a specific antibody-antigen interaction the B cell proliferates and generates clones engaged in *in vivo* selection and affinity maturation. The specificity of the V domains is conferred by the complementarity determining regions (CDR) and more particularly the CDR3 (19–21). The same features are observed in *in vitro* combinatorial libraries, which mimic the natural *in vivo* diversity, selection and affinity maturation (1–3).

In order to manage, analyze and compare the extraordinary diversity of the immune repertoires, IMGT^®^, the international ImMunoGeneTics information system^®^^1^ (22), was created in 1989 in Montpellier by Marie-Paule Lefranc (Montpellier University, CNRS), which marked the birth of immunoinformatics (18), a new science at the interface between immunogenetics and bioinformatics. IMGT^®^ has developed online tools that provide a detailed and accurate analysis of the V domains and which, in the case of nucleotide sequences, include IMGT/V-QUEST (23–25) for the analysis of the rearranged V-(D)-J sequences of the IG or antibodies and T cell receptors (TR), and IMGT/JunctionAnalysis (26, 27) for the analysis of the V-(D)-J junctions and of the included CDR3. The algorithms and IMGT reference directories of these tools have been implemented in IMGT/HighV-QUEST (28–31), the first and only web portal for NGS sequence analysis of IG and TR, begun in 2010. IMGT/HighV-QUEST analyses up to 500,000 NGS reads per batch and includes a statistical module for IMGT clonotype identification and comparison (analyses are performed on the results of up to one million reads, from one or several batches) (30). IMGT/StatClonotype (32, 33), a stand-alone tool and R package, allows for the comparison of IMGT clonotype diversity and expression between two NGS data sets, using the IMGT/HighV-QUEST statistical results output. In order to overcome the analysis challenge of the NGS scFv, the IMGT/V-QUEST functionality “Analysis of single chain Fragment variable (scFv)” which includes the search and characterization of two V domains in a single sequence [IMGT/V-QUEST Documentation^2^ (34)] has recently been integrated in IMGT/HighV-QUEST (IMGT/HighV-QUEST Documentation^3^).

The main challenge addressed in this study was to obtain high quality NGS reads of the scFv long enough to encompass the two VH and VL domains and to analyze and characterize the association of the two V domains in the NGS scFv reads using the scFv functionality in IMGT/HighV-QUEST. Full-length scFv reads are expected to have a length of around 800 bp with a whole insert of around 1,000 bp (including the 5′ and 3′ sequences of the vector and primer sequences at both ends). We used the Pacific Biosciences (PacBio) third-generation NGS technology, which provides long sequencing reads and the highest consensus accuracy available today (35–38). For this project, the high accuracy is a result of the generation of circular consensus sequencing (CCS) reads, by which the long sequencing reads allow for multiple passes of the same insert and thus removal of random sequencing errors upon consensus construction. Practically, one single molecule, real-time (SMRT) cell has 150,000 zero-mode waveguides (ZMWs), of which 50,000–75,000 are loaded with single molecules during sequencing, resulting in the production of ~50,000–75,000 unique consensus reads per run and per SMRT cell. PacBio NGS sequencing was performed from amplified DNA of scFv-phages isolated from the third round of an *in vivo* biopanning in an animal model of atherosclerosis (12, 13). The PacBio scFv CCS (version 2) reads were first analyzed using IMGT/HighV-QUEST with the scFv functionality. In a second step, the sequence quality was evaluated by tracking the NGS reads of a scFv-phage clone P3, identified by biological assays and Sanger sequencing.

## Materials and Methods

### *In Vivo* Selection of scFv-Phages Specific to Atheroma Plaque

**Figure 1:**
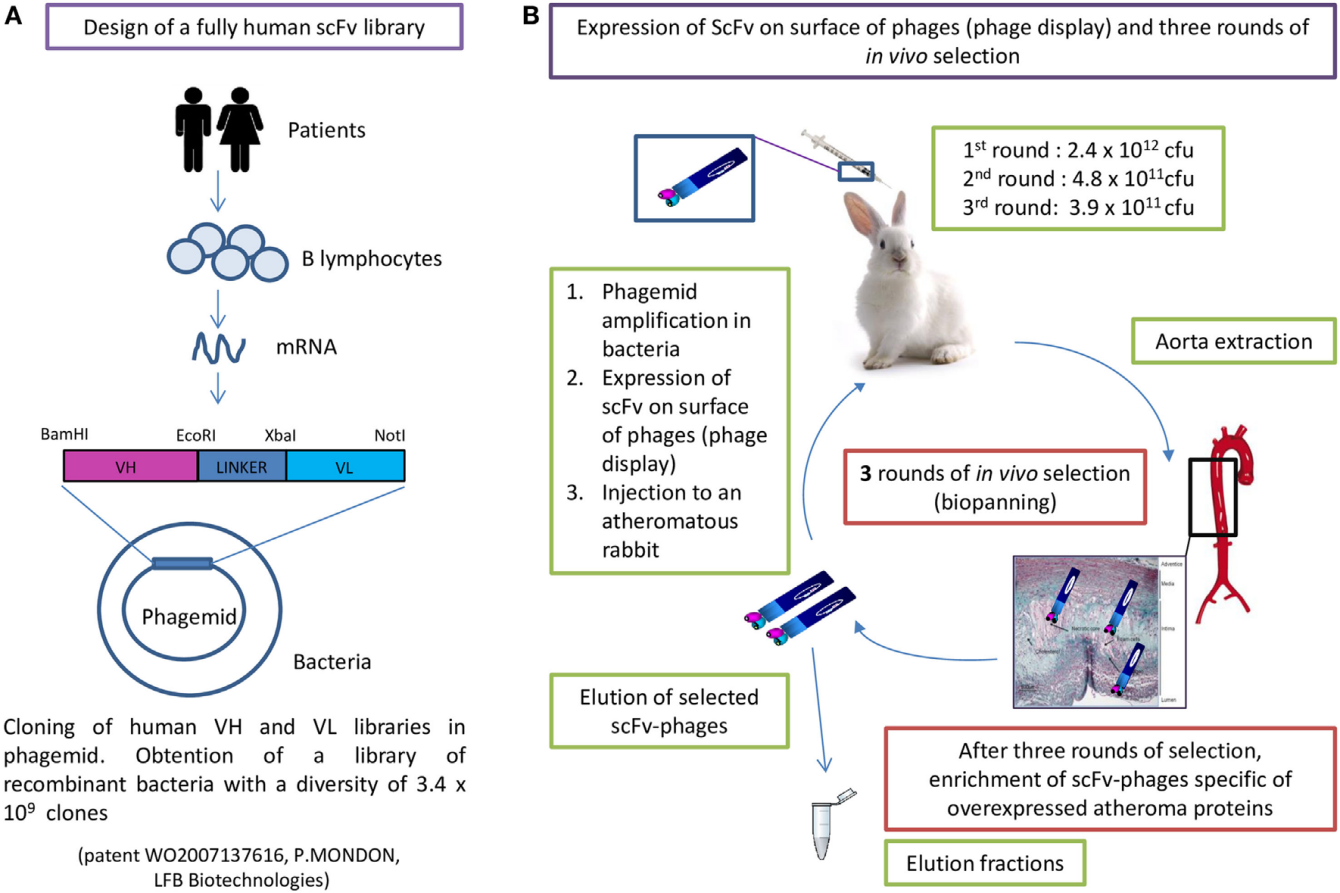
Human single chain fragment variable-phagemid combinatorial library construction **(A)** and *in vivo* phage display selection **(B)**.

A fully human-recombinant scFv antibody library (scFv cloned in the pMG72 phagemid vector, containing the ampicillin-resistant gene for the selection and maintenance of the phagemid) with a diversity of 3.4 × 10^9^ clones (full description in patent WO2007137616) was expressed by phage display and selected *in vivo*, as previously described (13). The scFv-phages were obtained from the scFv-phagemid combinatorial library by expression of the scFv on the phage surface, following the addition of a helper filamentous phage to recombinant phagemid infected bacteria in the exponential phase (39). Three rounds of biopanning were performed in atheromatous injured rabbits (12). All animal experiments were performed in conformity with the Guide for the Care and Use of Laboratory Animals (NIH Publication No. 85–23, revised 1996) and were accredited by the local ethical committee (Animal Care and Use Committee of Bordeaux, France under the No. 50120192). Briefly, the procedure was the following (Figure 1): 2.4 × 10^12^ colony-forming units (cfu) of scFv-phages were injected into an atheromatous rabbit. After 1 h in circulation, the animal was sacrificed, the aorta was retrieved and scFv-phages binding to the aorta were eluted in different fractions. The eluted scFv-phages were reamplified in XL1-Blue bacteria and following scFv expression at the phage surface as above (39), the amplified scFv-phages were reinjected in another atheromatous animal. Rounds 2 and 3 were conducted following the same procedure. The number of reinjected colony-forming units were 4.8 × 10^11^ in round 2 and 3.9 × 10^11^ in round 3. The total number of eluted scFv-phages from the third round was 1.5 × 10^7^ cfu (total for seven fractions corresponding to different areas of the aorta).

**Figure 2:**
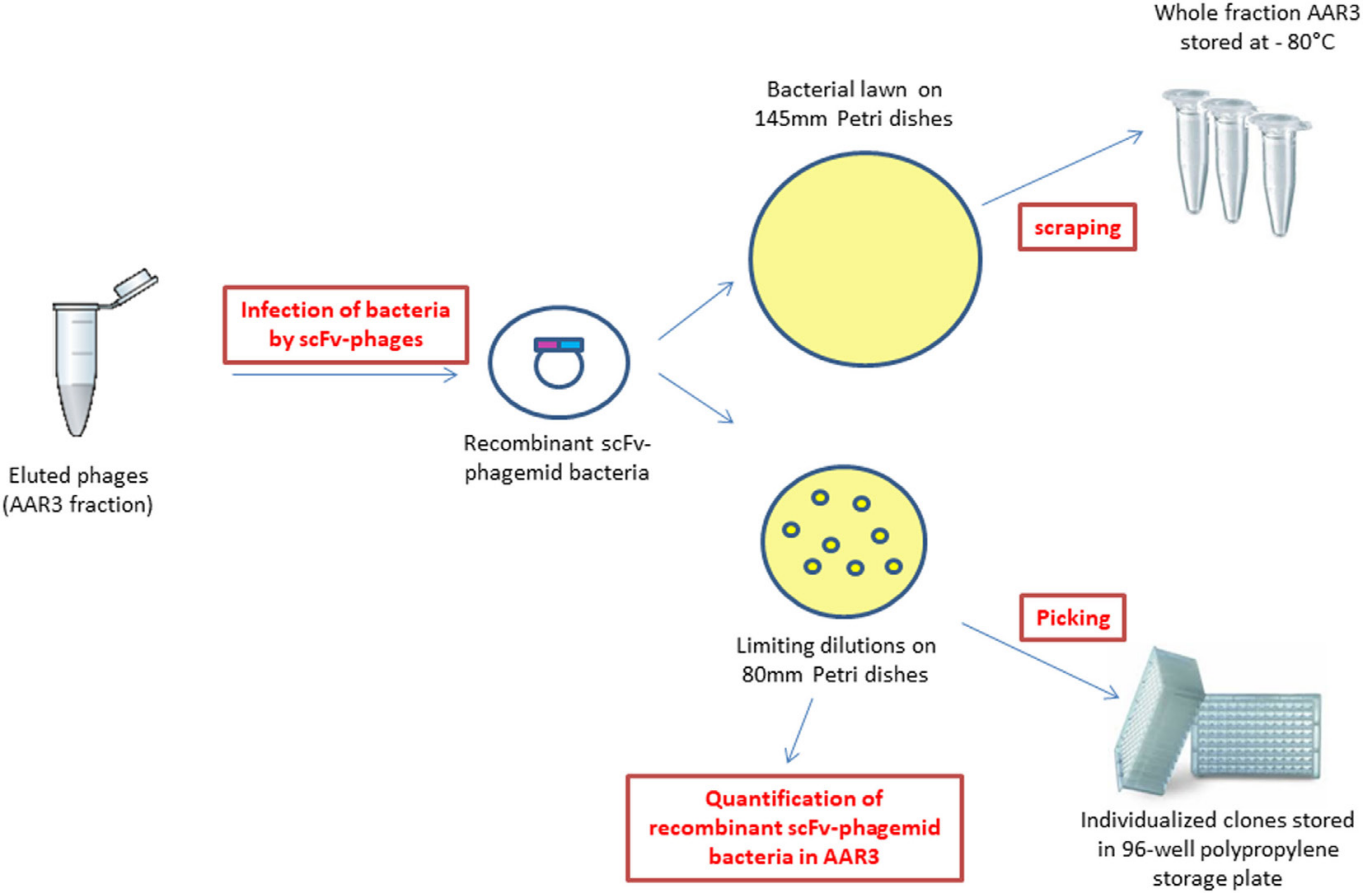
Obtaining recombinant single chain fragment variable-phagemid bacteria from the AAR3 fraction, storage for next-generation sequencing, quantification, and picking of individual clones.

In this study, the analyzed fraction is the one recovered after the third round of selection from the endothelial cells of the damaged abdominal aorta vessel wall (named AAR3 for abdominal aorta round 3) (Figure 2). The scFv-phages were amplified in XL1-Blue bacteria and plated on 145 mm Petri dishes for storage of the whole AAR3 fraction before sequencing, and on 80 mm Petri dishes for limiting dilution (quantification of recombinant bacteria and picking of individual clones) (Figure 2). The recombinant bacteria plated on 145 mm Petri dishes were scratched and stored at −80°C in 50% v/v glycerol. Around 3.5 × 10^5^ clones issuing from the whole AAR3 fraction were counted by limiting dilution of recombinant bacteria on 80 mm Petri dishes. Ninety-six recombinant bacteria clones were individually picked and grown in selective medium on a 96-well MASTERBLOCK^®^ polypropylene storage plate (Greiner Bio-One, France) and stored for *in vitro* bioreactivity assays (*in vitro* screening of scFv-phages on atheromatous proteins) and for Sanger sequencing.

Each of the 96 individual recombinant bacteria clones from the MASTERBLOCK^®^ was repicked on an agarose 96-Well plate (Beckman Coulter, France). After incubation at 37°C overnight, plasmid DNA extraction, PCR amplification and sequencing were then performed by Beckman Coulter (France). One of these sequenced clones, P3 (767 bp), was used in this study for tracking the PacBio reads that were identical or related to it.

### PacBio RS II Sequencing of the Whole AAR3 Fraction

#### Generating High Quality PCR Products

**Figure 3:**
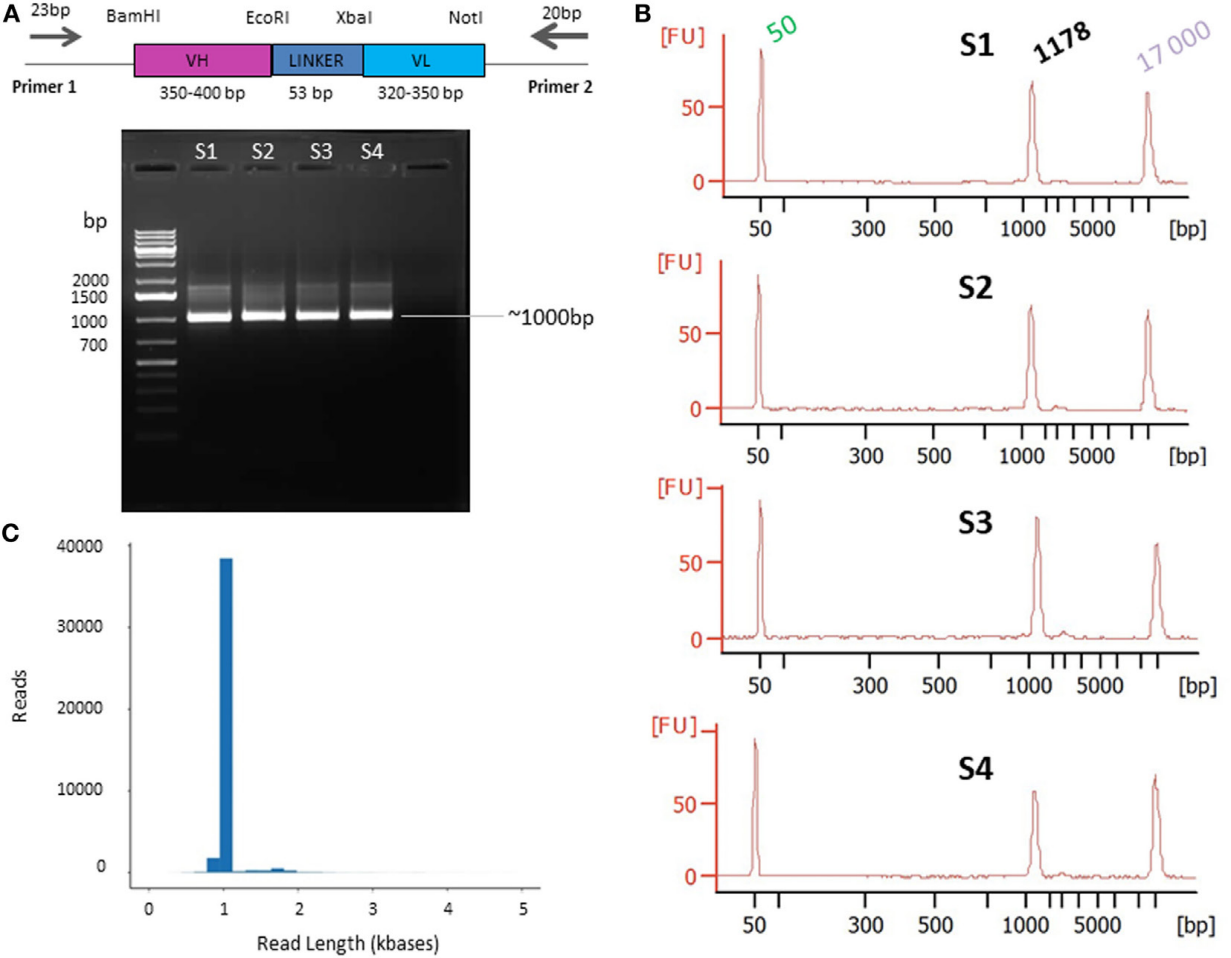
Primer design **(A)** and quality control presequencing **(B)** and postsequencing **(C)**. **(A)** Primers designed on the phagemid vector and used for single chain fragment variable (scFv) PCR amplification. The scFv (VH-LINKER-VL) length range is between ~720 and ~800 bp [variable heavy (VH) between ~350 and ~400 bp and variable light (VL) between ~320 and ~350 bp]. The linker is 53 bp including the EcoRI and XbaI sites. The PCR products are expected to be ~1,000 bp on average, including the 5′ and 3′ region and the primers. **(B)** Agarose gel electrophoresis of PCR products. The DNA was amplified from the AAR3 fraction and PCR products were analyzed on 1.2% (w/v) agarose gel. The band at ~1,000 bp corresponds to the expected size for scFv amplicons. S1, S2, S3, and S4 correspond to the samples 1, 2, 3, and 4, respectively. The Bioanalyzer trace of the four samples shows the purity of amplicons with a high-quality single peak. **(C)** Pacific Biosciences RS II CCS2 read length distribution using P6-C4 chemistry for 1 SMRT cell (similar results were obtained for the 15 SMRT cells). Data are based on a 1-kb size-selected scFv library using a 6 h movie.

Single-molecule real-time sequencing requires high-quality, doubled-stranded DNA as input. To ensure this, plagemid DNA of recombinant bacteria from the whole AAR3 fraction was extracted directly from the frozen extract just before PCR amplification, using the QIAprep spin miniprep kit (Qiagen, France) according to manufacturer’s instructions. To generate clean, undamaged and non-chimeric amplicons, the highest fidelity polymerase was used. All PCR reactions were performed in volumes of 50 µL using 25 µL of the KAPA HiFi™ HotStart ready Mix (Kapa Biosystems, France) and 20 ng of DNA template. Each primer was used at a final concentration of 0.3 µM. PCR reactions were performed with the forward primer (Primer 1, 23-mer FWD) 5′-TGCAAATTCTATTTCAAGGAGAC-3′ and the reverse primer (Primer 2, 20-mer REV) 5′-TCACGTGCAAAAGCAGCGGC-3′. These primers were designed based on the phagemid vector; for primer 1 from position −96 to −74 upstream of the BamHI site and for primer 2 from positions 95 to 114 downstream of the NotI site (Figure 3A).

The PCR cycling protocol was chosen according to the manufacturer’s instructions and consisted of 95°C for 3 min; 15 cycles of 98°C 20 s, 65°C 15 s, 72°C 30 s; one 72°C 1 min followed by one cycle at 4°C. The reduction of the number of PCR cycles minimizes the PCR bias, however when further decreases in the number of PCR cycles were attempted, it led to several contaminating, off-target bands on an agarose gel.

The required quantity in PacBio guidelines for insert sizes superior to 750 bp is 500 ng of DNA. Four PCR reactions were performed on the same AAR3 fraction for reproducibility purposes. Agarose gel electrophoresis was used to confirm amplification, correct fragment size, and to check for non-specific product contamination. A sizing marker was included to confirm size specificity (GeneRuler 1 kb Plus DNA Ladder, Thermo Fisher Scientific, France). Amplicons were cleaned using 1× ratio of AMPure PB Beads (Pacific Biosciences). DNA purity and quantification (sample volume, yields and size distributions) were evaluated and measured using the Agilent 2100 Bioanalyzer DNA12000 kit (Agilent technologies).

#### SMRTbell Template Preparation

SMRTbell templates (PacBio, CA, USA) were constructed following the standard Amplicon Sequencing Protocol.^4^ The full procedure is explained in Figure S1 in Supplementary Material.

Briefly, PCR products were treated to repair DNA damage and then hairpin adapters were added *via* blunt end ligation to produce SMRTbell templates using the SMRTbell template prep kit 1.0. Exonucleases III and VII were used to remove failed ligation products and SMRTbell templates were purified with AMPure PB Beads. The ratio of sequencing primer and polymerase was determined by a PacBio calculator to correlate with SMRTbell concentrations and the 1,100-bp insert size. The sequencing primer was annealed to the single-stranded loop region of the SMRTbell template, and primer-annealed templates were then bound to DNA polymerase P6 using the DNA/polymerase binding kit P6v2. The DNA–polymerase complexes were loaded on 15 SMRT cells using MagBeads onto the PacBio RS II system and sequenced using the C4 chemistry and 6-h movies.

Initial loading titrations were performed to identify the optimal loading concentration, identifying 0.03–0.035 nM as the best loading conditions for scFv PCR products. Each SMRT cell generates ~50,000 reads on average (considering that 50,000–75,000 ZMW can be optimally loaded with a single molecule of DNA). A total of 15 SMRT cells were loaded for the four different PCR products of the same AAR3 fraction (three SMRT for sample 1 and four SMRT for samples 2, 3, and 4) to cover, per PCR sample, the diversity of the AAR3 fraction (Figure S2 in Supplementary Material). The use of 15 SMRT cells was chosen to provide a thorough and sound proof-of-concept and read comparison from four different PCR amplicons generated from the same fraction (AAR3).

#### PacBio CCS Read Generation

The PacBio RSII instrument produces sequencing reads with an average read length of ~15 kb, which would theoretically pass over a 1,100 bp molecule more than 10 times, producing CCS2 sequences with an accuracy ~99.9%. Longer read lengths can be achieved by increasing the instrument run time, so these data were collected using 15 SMRT cells and 6 h movies, to maximize read length and, thus, number of passes. Following sequencing, the raw data were processed using the CCS2 pipeline (CCS code used available at https://github.com/PacificBiosciences/unanimity). All CCS2 reads that were 99.9% accurate or greater were exported for further analysis. The raw NGS data of the 85-related P3 sequences can be found in the NCBI Sequence Read Archive with the accession number SRP124616.

### IMGT/HighV-QUEST Analysis of the PacBio CCS2 Reads

#### Characterizing IMGT/HighV-QUEST scFv Reads from AAR3

The FASTQ files of the PacBio CCS2 reads were imported and converted to FASTA sequences for submission to IMGT/HighV-QUEST^5^ (29, 30), which implements IMGT/V-QUEST program version 3.4.2 (August 4, 2016) and IMGT/V-QUEST reference directory release 201631-4. The analysis was performed with the advanced functionality “Analysis of single chain Fragment variable (scFv)” (IMGT/HighV-QUEST Documentation, see text footnote 3).

Data filtering was applied with the following criteria to be fulfilled for each of the two V domains: (i) >85% of identity of the V-REGION of the V domain with the V-REGION of the closest germline IMGT gene and allele and (ii) in-frame V-(D)-J junction. Filtered sequences were then analyzed to identify the closest V, D (for VH) and J IMGT genes and alleles, to characterize the amino acid (AA) junction, to evaluate the mutations and to give a complete description of the scFv with IMGT labels (IMGT Index > scFv^6^).

#### Tracking and Analysis of Identical and Related PacBio Reads of the AAR3 scFv-Phage Clone P3

In order to evaluate the PacBio scFv read sequencing quality, reads identical or closely related to the sequence of a scFv-phage clone P3 (previously isolated from the same fraction AAR3 and Sanger sequenced) were tracked among the total reads generated in the 15 SMRT cells by two approaches. First, reads potentially related to P3 were searched for based on the expected VH-VL characteristics determined by IMGT/HighV-QUEST (same V and J genes and alleles, and same AA junctions). Second, a Blast search was performed to check whether P3-related reads could have been missed by the IMGT/HighV-QUEST filtering. The fasta headers of the sequences were modified to include the sequence set identifier and a blast database was built (formatdb 2.2.26) from the accumulated reads from the 15 SMRT cells. The database search was performed using the blastn program (2.2.26), with the combined following criteria: longest alignments on the P3 Sanger (767 bp) sequence, highest identity percentage, and maximum number of 20 mismatches or indels. Among the extracted reads only those fulfilling the P3 characteristics (in terms of V and J genes, allele names and AA junctions) were retained.

## Results

### Generation and Analysis of PCR Products from the Whole AAR3 Fraction

The unbiased characterization of scFv from phage-display combinatorial libraries, in conjunction with sequencing on the PacBio RS II system, requires high-quality PCR products with undamaged, clean and non-chimeric amplicons. Creating PCR protocols to generate products that are truly representative of the starting cell population is a major challenge.

To achieve this aim: (1) the complete frozen AAR3 fraction was directly used, without amplification, as the source of recombinant bacteria; (2) efforts were made to limit the number of amplification cycles (the four samples subject to 15 cycles) in order to reduce quantitative distortions as well as error rates due to PCR artifact (data not shown); and (3) different high-fidelity Taq polymerases were tested so as to fit with our amplification system. The required quantity (500 ng) and the correct size of amplicons (~1,000 bp) were obtained with the KAPA HiFi™ HotStart polymerase (Figure 3A). The quality and quantity of amplicons was confirmed using an Agilent 2100 Bioanalyzer. After confirmation of the purity (Figure 3B), PCR samples were sequenced on the PacBio platform.

### PacBio Sequencing and CCS2 Analysis

Sequencing was done on the PacBio RS II system using P6-C4 sequencing chemistry and SMRTbell libraries generated from DNA amplified from the AAR3 fraction (corresponding to 3.5 × 10^5^ scFv-phagemids). For each of the four PCR samples, 3 or 4 SMRT cells were run to ensure adequate sampling of the scFv as described in Section “Materials and Methods” (Figure S2 in Supplementary Material). A total of 15 SMRT cells were used.

Circular consensus sequencing (CCS2) analysis of the 15 SMRT cells produced 450,558 reads. These reads were filtered to remove any double-loaded wells or other artifactual/lower accuracy reads. The data obtained from filtered, post-CCS2 analysis represent the reads achieving 99.9% accuracy or above, derived only from wells loaded with a single molecule. Any reads that did not reach consensus coverage of QV30 (99.9% accurate) or above were filtered out. Those settings and filters are built into the CCS2 pipeline that is available *via* the PacBio web-based analysis software (SMRT Link). Thus, these 450,558 reads passed initial filtering with an average pass number of 24 and a quality score of minimum 99.94% accuracy (Figure 3C). Another contributing factor to data quality and throughput was the use of the longest movie lengths possible at the time (6 h) to ensure the longest read lengths for analysis.

### IMGT/HighV-QUEST Analysis of the scFv PacBio Reads

The 450,558 FASTQ PacBio CCS2 reads were converted in FASTA sequences and analyzed using IMGT/HighV-QUEST, as described in Section “Materials and Methods.” After analysis, a total of 391,655 scFv “candidates” (i.e., sequences with two V regions, IMGT label V-REGION) (86.93% of the submitted PacBio reads) were identified (Table 1). The scFv sequences were then filtered according to the criteria described in Section “Materials and Methods” [>85% of identity of the V-REGION of the V domains with the V-REGION of the closest germline IMGT genes and alleles and in-frame V-(D)-J junction, determined for both V domains]. The threshold of 85% of identity is the standard filter for classical IG repertoire analysis in IMGT/HighV-QUEST (29). Following this filtering, 348,659 full-length scFv representing 89.02% of the filtered sequences were identified (Table 1). These scFv reads include 346,934 VH-VL or VL-VH expected scFv sequences (Table S1 in Supplementary Material). The other 1,725 scFv reads comprise 171 VH-VH (5–22 found per SMRT cell) and 1,554 VL-VL (68–158 found per SMRT cell). These combinations most probably occurred during the construction of the original scFv-phage combinatorial library. Thus these results provide a useful and detailed overview of the content of the scFv combinatorial library.

**Table 1:** IMGT/HighV-QUEST analysis of the scFv Pacific Biosciences (PacBio) reads.

PCR sample no.	Number of reads with 99.9% predicted accuracy	Mean number of passes	Number of movies	SMRT cell no.	Number of PacBio CCS2 analyzed reads	Number of scFv candidates in analyzed reads	% of scFv candidates in analyzed reads, i.e., coverage	Number of filtered-in reads	% of scFv in filtered-in reads, i.e., coverage	% of scFv in analyzed reads, i.e., coverage
s1	91,828	24	3	1	29,224	25,419	86.98	22,906	90.11	78.38
				2	32,240	28,120	87.22	25,228	89.72	78.25
				3	30,364	26,496	87.26	23,799	89.82	78.38

s2	129,640	23	4	4	34,082	29,729	87.23	26,657	89.67	78.21
				5	33,510	29,213	87.18	26,407	90.39	78.80
				6	31,980	27,874	87.16	25,032	89.80	78.27
				7	30,068	26,289	87.43	23,695	90.13	78.80

s3	115,446	24	4	8	34,890	30,183	86.51	26,990	89.42	77.36
				9	29,373	25,314	86.18	22,468	88.76	76.49
				10	26,465	22,776	86.06	20,044	88.00	75.74
				11	24,718	21,358	86.41	18,741	87.75	75.82

s4	113,644	24	4	12	25,128	21,881	87.08	19,120	87.38	76.09
				13	23,693	20,515	86.59	17,756	86.55	74.94
				14	32,293	28,093	86.99	24,762	88.14	76.68
				15	32,530	28,395	87.29	25,054	88.23	77.02

Total	450,558				450,558	391,655	86.93	348,659	89.02	77.38

*Each CCS read counts as “1× coverage” over the scFv molecules of interest. The coverage is given in percentage of the scFv of interest (analyzed reads, filtered-in reads and analyzed reads)*.

The IMGT/HighV-QUEST analysis of the scFv reads included identification of the closest V, D (if VH), and J IMGT genes and alleles, characterization of the junction, evaluation of the mutations, and complete description of both V domains with IMGT labels (see text footnotes 2 and 5). These results demonstrate that both domains of the scFv reads sequenced by PacBio could be fully characterized with the functionality for scFv.

### Tracking and Analysis of PacBio Reads Identical or Related to the P3 Clone across 15 SMRT Cells

Single chain fragment variable-phages issuing from the *in vivo* AAR3 selected fraction have been screened by a high-throughput flow cytometry method against atherosclerotic rabbit proteins. Some of the selected clones were then processed into scFv-Fc format in HEK293 cells and validated by immunohistochemistry on atheromatous aorta sections of two animal models of atherosclerosis (ApoE^−/−^ mouse and New Zealand White rabbit submitted to hypercholesterolemic diet) and on human endarteriectomy biopsies. A scFv-phage clone, named P3, selected for its high accuracy in biological assays in the two animal models and human biopsies was Sanger sequenced and patented (EP 17306337.1). The whole scFv P3 sequence (Figure S3 in Supplementary Material) was then tracked among the 346,934 VH-VL and VL-VH reads (Table S1 in Supplementary Material) from the 15 SMRT cells, by the two approaches described in Section “Materials and Methods,” in order to evaluate the PacBio scFv read sequencing quality.

#### Analysis of PacBio Reads Identical to the P3 Clone

Sixty PacBio reads with 100% identity to the region aligned with the P3 Sanger sequence (767 bp) were obtained by both approaches. Interestingly, the P3 scFv was identified in the top 100 of the most abundant VH-VL associations in all the 15 data sets. These PacBio reads have a length of 977 bp, including the 5′ and 3′ regions and the primers. Fifty-three of the 60 reads have a 100% identity on their full length of 977 bp. As these PacBio reads were obtained from the 15 SMRT cells (Table 2), we can confidently say that no sequencing error was observed in the 51,781 bp of these 53 reads.

**Table 2:** Pacific Biosciences (PacBio) reads 100% identical to the aligned P3 Sanger sequence and 100% identical between them on 977 bp (53 reads) or 939 bp (7 reads).

PCR sample no.	Number of P3 PacBio reads per PCR sample	SMRT cell no.	Number of P3 PacBio reads per SMRT cell	100% on 977 bp (53 reads)	100% on 939 bp (7 reads)	GenBank/ENA/DDBJ accession number
s1	15	1	6	1, 2, 3, 4, 6, 8		MG272208
		2	5	10, 30	11, 12, 13	
		3	4	16, 17, 19	15	

s2	14	4	2	32, 33		
		5	2	36	35	
		6	3	37, 38, 39		
		7	7	20, 41, 44, 45, 46, 47, 48		

s3	15	8	6	49, 50, 51, 52, 53, 54		
		9	4	56, 57, 59, 60		
		10	4	63, 65, 66	64	
		11	1	68		

s4	16	12	4	70, 72, 73	71	
		13	2	75, 76		
		14	3	21, 80, 83	
		15	7	23, 24, 25, 26, 27, 28, 29	

Total	60		60	53	7

Seven reads showed 100% identity on a 939 bp length (Table 2). Interestingly their mutations are all localized at the ends of the primers and/or in the immediate vicinity of the 3′ primer (Table 3). A most probable explanation is that they occur during the sequencing polymerization (the priming step could be excluded as different mutations were observed in different SMRT cells and no degenerate bases were used in the primers). Ignoring the mutations in or next to the primers, no sequencing error was observed in 6,573 nucleotides of the 7 reads (100% identity on 939 bp). Combining these results with those of the 53 reads (100% identity on the full length of 977 bp), no sequencing error was detected on 58,354 nucleotides for the 60 scFv reads.

**Table 3:** Mutations observed at the 5′ and 3′ end of the seven Pacific Biosciences (PacBio) reads with 100% identity on 939 bp (positions 3–941).

PacBio read no. (assigned in the list 1–85)	PCR sample no.	SMRT cell no.	Mutation description^a^	Mutation localization	GenBank/ENA/DDBJ accession number
13	s1	2	One 1 nt-deletion (g2 > del)	5′ end of the 5′ primer	MG272209

11	s1	2	Two 1 nt-deletion (t975 > del, a977 > del)	3′ end of the 3′ primer	MG272210

15	s1	3	One 1 nt-substitution (c956 > t)	Vicinity of the 3′ primer	MG272211

12	s1	2	One 1 nt-deletion (a942 > del)^b^	Vicinity of the 3′ primer^b^	MG272212
35	s2	5			
71	s4	12			

64	s3	10	Two 1 nt-deletion (a942 > del),^b^ (t975 > del)	Vicinity of the 3′ primer,^b^ 3′ end of the 3′ primer	MG272213

*Positions of the primers are 1–23 and 958–977*.

*^a^Mutations are described according to the IMGT Scientific chart rules (http://www.imgt.org/IMGTScientificChart/Nomenclature/IMGTmutation.html) (40)*.

*^b^The 1 nt-deletion (a942 > del) found in reads from the four samples most probably originates from the library*.

#### Analysis of PacBio CCS Reads Related to the P3 Clone

In addition to the 60 PacBio reads with a 100% identity (53 reads on 977 bp and seven reads on 939 bp) (Table 2), 25 “related” P3 reads were identified (Figure S4 in Supplementary Material). These 25 P3-related reads showed 15 different mutation types in the insert and, for each type, a limited number of reads (1–6) (Table 4). Altogether, the 15 mutation types are described by 28 different individual mutations. This heterogeneity is in sharp contrast with the 60 reads with a 100% identical insert.

**Table 4:** Mutation heterogeneity observed in 25 P3-related PacBio reads, in contrast with the 60 P3 identical PacBio reads with 100% identity on the complete scFv.

Read categories	Pacific Biosciences (PacBio) read no. (assigned in the list 1–85)	PCR sample no.	SMRT cell No.	Number of reads/mutation type	Mutation type	Mutation description^a^	GenBank/ENA/DDBJ accession number
A (15 reads)	7, 18, 43, 67, 69, 79	1, 2, 3, 4	1, 3, 7, 11, 12, 14	6	Four 1 nt-substitution	a545 > g (VL), g686 > a (VL), a757 > g (VL), c838 > g (VL)	MG272218

	58	3	9	1	Four 1 nt-substitution with, in 3′, a large deletion	a545 > g (VL), g686 > a (VL), a757 > g (VL), c838 > g (VL), a886-a977 > del (92 nt)	MG272219

	61	3	9	2	Four 1 nt-substitution	c741 > t (VL), g837 > a (VL), c838 > g (VL), g843 > t (VL)	MG272220
		
	74	4	13		Four 1 nt-substitution *with, at the 3*′ *end of the 3*′ *primer, a 1 nt- deletion*	c741 > t (VL), g837 > a (VL), c838 > g (VL), g843 > t (VL), *a977* > *del*	MG272221

	5, 14, 31, 34, 42, 82	1, 2, 4	1, 2, 4, 7, 14	6	Two 1 nt-substitution	c720 > t (VL), t744 > c (VL)	MG272223

B (10 reads)	85	2	4	2	One 1 nt-deletion	c242 > del (VH)	MG272216
	40	2	6		One 1 nt-deletion	g600 > del (VL)	MG272217

	77	4	13	6	One 1 nt-substitution	g495 > a (linker)	MG272227
	22	4	14		One 1 nt-substitution	t624 > c (VL)	MG272224
	55	3	9		One 1 nt-substitution	a627 > g (VL)	MG272225
	62	3	10		One 1 nt-substitution	g736 > a (VL)	MG272226
	78	4	13		One 1 nt-substitution *with, at the 3*′ *end of the 3*′ *primer, a 3 nt-deletion*	t599 > g (VL), *t975-a977* > *del (3nt)*	MG272228
	81	4	14		One 1 nt-substitution *with, at the 5*′ *end of the 5*′ *primer, a 1 nt-deletion, and at the 3*′ *end, a 19-nt primer deletion*	*g2* > *del*, a715 > g (VL), *c959-a977* > *del (19 nt)*	MG272222

	84	2	5	1	Two 1 nt-insertion	209^210 > ins^a (VH), 762^763 > ins^t (VL)	MG272214

	9	1	2	1	One 1 nt-substitution in VH, one 2 nt-insertion + two 1 nt-substitution in VL	c322 > t (VH), 658^659 > ins^cc (VL), c659 > t (VL), t660 > a (VL)	MG272215

Total: 25				Total: 25			

*Positions of the primers are 1–23 and 958–977. Category A: 15 P3-related reads of potential biological interest (mutations due to the VL diversity originating from the combinatorial library). Pink, green, and blue colors highlight groups of reads with in common identical substitution mutations. Category B: 10 P3-related reads with undefined origin of the mutations*.

*^a^Mutations are described according to the IMGT Scientific chart rules (http://www.imgt.org/IMGTScientificChart/Nomenclature/IMGTmutation.html) (40). The mutations in the primers are shown in italics*.

The observation of related reads was expected as the scFv are from a phagemid combinatorial library in which point mutations were initially introduced to mimic the IG somatic hypermutations. The analysis of the 25 reads was therefore performed in an attempt to distinguish among them related clones with potential biological interest (as reflecting the diversity of the original library) from reads with artifactual differences.

For 15 of these PacBio CCS reads, the origin of the mutations (mostly substitutions) was clearly from the scFv phagemid combinatorial library (and therefore of potential interest given their relatedness to P3). This was supported by the fact that identical mutated reads were obtained from different PCR and from different SMRT cells (category A in Table 4). These included 7 reads (highlighted in pink) with four identical individual mutations, 2 reads (highlighted in green) with three identical individual mutations different from those described above and one shared by both groups [c838 > g (VL)], 6 reads (highlighted in blue) with two identical individual mutations (and still different from those of the previous mutation types). All the substitution mutations are localized in the VL, in agreement with mutations of the VL and VH domains being targeted differently before the assembly into the scFv and confirming that the differences observed are intrinsic to the VL domain, and not due to PCR or sequencing errors. The IMGT/V-QUEST alignment of the 15 PacBio NGS sequences with the initial P3 Sanger sequence is provided as an additional PDF file in supplementary data (Figure S5 in Supplementary Material).

For the other 10 PacBio reads, present in one copy (category B in Table 4), the origin of the differences could not be determined with certainty: and any of the possible explanations: putative sequencing errors, PCR amplification errors or diversity of the combinatorial library could not be formally excluded at this stage. The 1 nt-deletion observed in VH and VL for reads 85 and 40, respectively, the large nt deletion at the 3′ end in read 78, the nt deletion at both 5′ and 3′ ends for read 81 and the one or two nt insertions in VH or VL for reads 84 and 9 could be considered as sequencing errors. The six reads with different single substitution and the one with 3 mutations could be attributed to PCR amplification errors or diversity of the combinatorial library. Moreover, and even if these differences are due to diversity of the combinatorial library, their single copy number suggests that they are from unselected (or poorly selected) scFv-phages from the library background and could be ignored in the library screening.

## Discussion

Antibody libraries are important resources to derive antibodies to be used for a wide range of diagnostic and therapeutic applications. *In vivo* or *ex vivo* phage-display selections have emerged as interesting ways to identify accurate antibodies in the context of the pathologic microenvironment (11). Although advancements in automation of biological assays have greatly improved screening strategies, high-throughput campaigns are still severely limited in the number of antibody fragments that can be interrogated, providing only a tiny fraction of the enriched phage library. Access to the genetic information encoded in antibody repertoires by NGS should allow more in depth analysis of the diversity of the selected library. However, the currently available NGS platforms that were capable of providing several million reads per run, generated only short reads of up to 300–700 bp (41). Therefore, only synthetic combinatorial scFv libraries, in which diversity was confined to CDR3 regions of the heavy and light chains, have benefited from NGS potential in the extensive *in silico* analysis of complex collections of selected antibodies (16).

While Mi-Seq, 454, or Ion-Torrent technologies will completely sequence heavy and light variable domains, they are currently insufficient to cover the full-length scFv, which are comprised of both VH and VL domains, connected by a peptide linker. These technologies often necessitate consensus building of multiple reads originating from the scFv fragment to obtain whole sequence information (42–44). Therefore, NGS sequencing of scFv fragments longer than 800 bp is still hampered by technical limitations in the length of reads. These limitations could be circumvented by the third generation PacBio sequencing platform. Capitalizing on PacBio SMRT DNA sequencing for high-resolution and high-throughput HLA typing (36, 37), we develop here a PacBio SMRT/CCS2 approach, combined with IMGT/HighV-QUEST analysis of full-length scFv reads to provide a straightforward protocol for characterization of the complete VH and VL domains of scFv from fully human combinatorial libraries.

Pacific Biosciences SMRT sequencing generated 450,558 reads of about 1,000 bp across 15 SMRT cells, following DNA amplification of the scFv insert from 3.5 × 10^5^ *in vivo* selected scFv-phages. IMGT/HighV-QUEST with its scFv functionality allowed for further filtration and characterization of the 348,659 PacBio CCS reads as containing full-length scFv, which represent 89.02% of the overall filtered sequences from the 15 SMRT cells run. Among them, 346,934 identified expected VH-VL or VL-VH full-length scFv reads.

In order to evaluate the PacBio scFv read sequencing quality, a selected scFv-phage clone P3, previously identified from the AAR3 fraction by biological screenings and sequenced by the Sanger methodology, was tracked within the 346,934 VH-VL and VL-VH scFv reads characterized by IMGT/HighV-QUEST. Sixty PacBio CCS2 reads were identified from the 15 SMRT cells that demonstrated 100% identity on a length of 939 bp (including the complete scFv of 767 bp and the 5′ and 3′ regions) and for 53 of them on the full length of 977 bp (including the primers at both ends). Thus no sequencing error was observed on a total of 51,728 bp for these 53 reads, obtained from the 15 SMRT cells (and 58,354 bp if the seven reads with 100% on 939 bp are included), validating the capacity of the PacBio SMRT-CCS2 method to produce reads with an accuracy of >99.9% for complete sequences of inserts approaching 1,000 bp. It is of interest to note that these PacBio reads were obtained from the 15 SMRT cells.

In addition to the 60 PacBio reads with a 100% identity, 25 “related” P3 reads were identified and for 15 of them, mutation analysis (category A in Table 4) clearly showed that they are related to P3. This was supported by the fact that identical mutated reads in the VL (in agreement with the diversity generated during the library construction) were obtained from different PCR samples and from different SMRT cells. P3 (60 reads) with its 15 related reads are among the top 100 most represented associated VH-VL domains in all the 15 data sets.

This study provides the first proof-of-concept that similar sequences could be tracked in phage-display selected scFv samples and their frequency determined by the number of reads. The favorite candidates chosen for their high frequency of enrichment could be rescued from these *in silico* data for implementation of downstream biological assays. This could be easily done by custom gene chemical synthesis, which offers the utmost flexibility and efficiency with high production yields (45).

Thus the large number of sequenced reads delivered, following the enrichment process, could be ideally suited for a more extensive evaluation of antibody candidates by biological assays. In that way, bringing full sequence data from NGS will accelerate search for identification of both the antibodies and their targeted biomarkers. We thus aimed to combine the sensitivity of the sequencing approach with the functional information provided by the immune assays. This *in silico* approach could be applied to any other pathology and phage-display screening methodology. Resolving the issue of complete scFv sequencing has the potential to profoundly impact the selection process of antibodies with desired properties after phage display biopanning with a special focus of *in vivo* selections. It is expected that this will contribute to therapeutic antibody discovery, selection and development.

### Limitations

Scientists working on IGs are very concerned by the problems of sequencing errors versus mutations and of short read assembly. This study demonstrates that the PacBio SMRT-CCS2 method is able to produce reads with an accuracy of >99.9% for complete sequences of scFv inserts without the need of *in silico* VH and VL assembly as usually necessary using MiSeq or Ion Torrent technologies (43, 44). To assess the read quality of PacBio sequencing, we performed a pilot study using different PCR samples and 3 or 4 SMRT for each PCR, starting from the same AAR3 fraction. Using the biologically validated P3 clone as a reference, we demonstrated that P3 clone is among the top 100 *in vivo* selected clones with a representativity of 0.025%. Moreover, among the 85 P3 related sequences, 60 were 100% identical and 15 clearly originated from the scFv phagemid combinatorial library. From the IMGT/V-QUEST alignment of P3 pink, blue, and green groups of reads with the initial P3 Sanger sequence, we can confidently say that 88% of reads were free of sequencing errors. However, a doubt remains for 10 of them, present in just one copy. The nt-deletions or nt insertions observed in VH and VL for six of the 10 reads could be definitively considered as sequencing errors. For the single substitutions observed in seven reads, it is obviously impossible to determine their origin. To circumvent this limitation, other technologies could be considered, such as inverse PCR method based on VH CDR3 or VL CDR3 sequences (44) to synthesize these clones from the AAR3 fraction. Nonetheless, what is of upmost importance in our study is to identify over-represented clones (from the top 100 candidates) and to proceed to the rescue of highly enriched scFv and not isolated clones. Indeed, during phage-display selections, the reads of greatest interest will have the greatest depth of coverage, having expanded in the pool and thus receiving greater proportional read depth.

## Ethics Statement

All animal experiments were performed in conformity with the Guide for the Care and Use of Laboratory Animals (NIH Publication No. 85–23, revised 1996) and were accredited by the local ethical committee (Animal Care and Use Committee of Bordeaux, France under the No. 50120192).

## Author Contributions

AH, FO, M-JJ-V, JL-T, MS, SK, M-PL, and GC-S conceived and designed the experiments. PM provided the library. MS sequenced the data. NH processed the raw data. VG designed the analysis algorithms and PD implemented the tool for NGS. CH, JKuijpers, AG, and JKorlach supervised the sequencing procedure. GC-S and M-PL supervised the project. AH, MS, GC-S and M-PL wrote the article. All the authors have read and approved the final manuscript.

## Conflict of Interest Statement

CH, NH, JKuijpers, and JKorlach are full-time employees at Pacific Biosciences, a company developing single-molecule sequencing technologies. MS was a full-time employee at Pacific Biosciences at the time of this work. PM is full-time employee at LFB, a pharmaceutical group specializing in biological medicinal products. The other authors declare that they have no conflict of interest.

## Footnotes

^1^http://www.imgt.org.

^2^http://www.imgt.org/IMGT_vquest/share/textes/imgtvquest.html.

^3^http://www.imgt.org/HighV-QUEST/doc.action.

^4^http://www.pacb.com/wp-content/uploads/Procedure-Checklist-Amplicon-Template-Preparation-and-Sequencing.pdf.

^5^http://www.imgt.org/HighV-QUEST/login.action.

^6^http://www.imgt.org/IMGTindex/scFv.php.

## Abbreviations

CCS, circular consensus sequencing; IG, immunoglobulin; IMGT, IMGT^®^, the International ImMunoGeneTics Information System^®^; NGS, next-generation sequencing; scFv, single chain Fragment variable; SMRT, single-molecule real-time; TR, T cell receptor.
