# Supplementary figures and images for: Pacific Biosciences Sequencing and IMGT/HighV-QUEST Analysis of Full-Length Single Chain Fragment Variable from an In Vivo Selected Phage-Display Combinatorial Library
Source: Front Immunol. 2017 Dec 20;8:1796. doi: 10.3389/fimmu.2017.01796 (PMC5742356; doi:10.3389/fimmu.2017.01796)

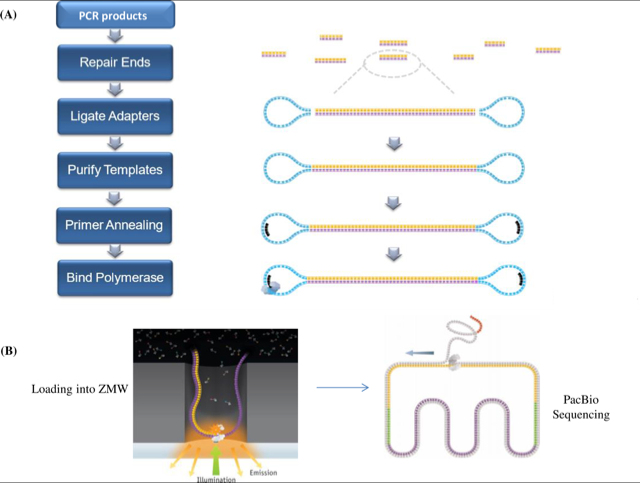

Supplement: Supplementary file 1 [file figure_s1.tif]

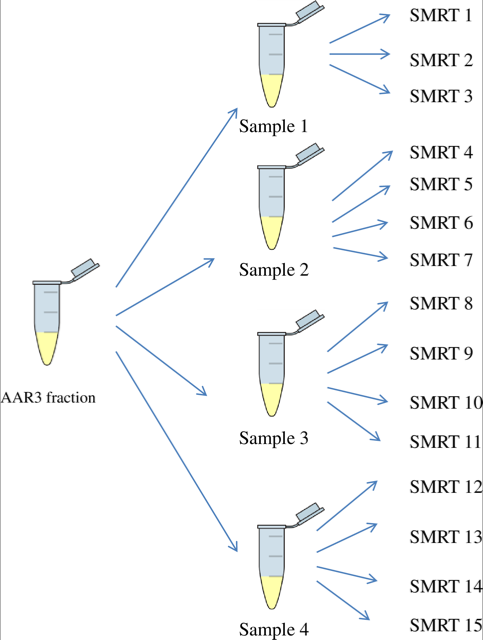

Supplement: Supplementary file 2 [file figure_s2.tif]

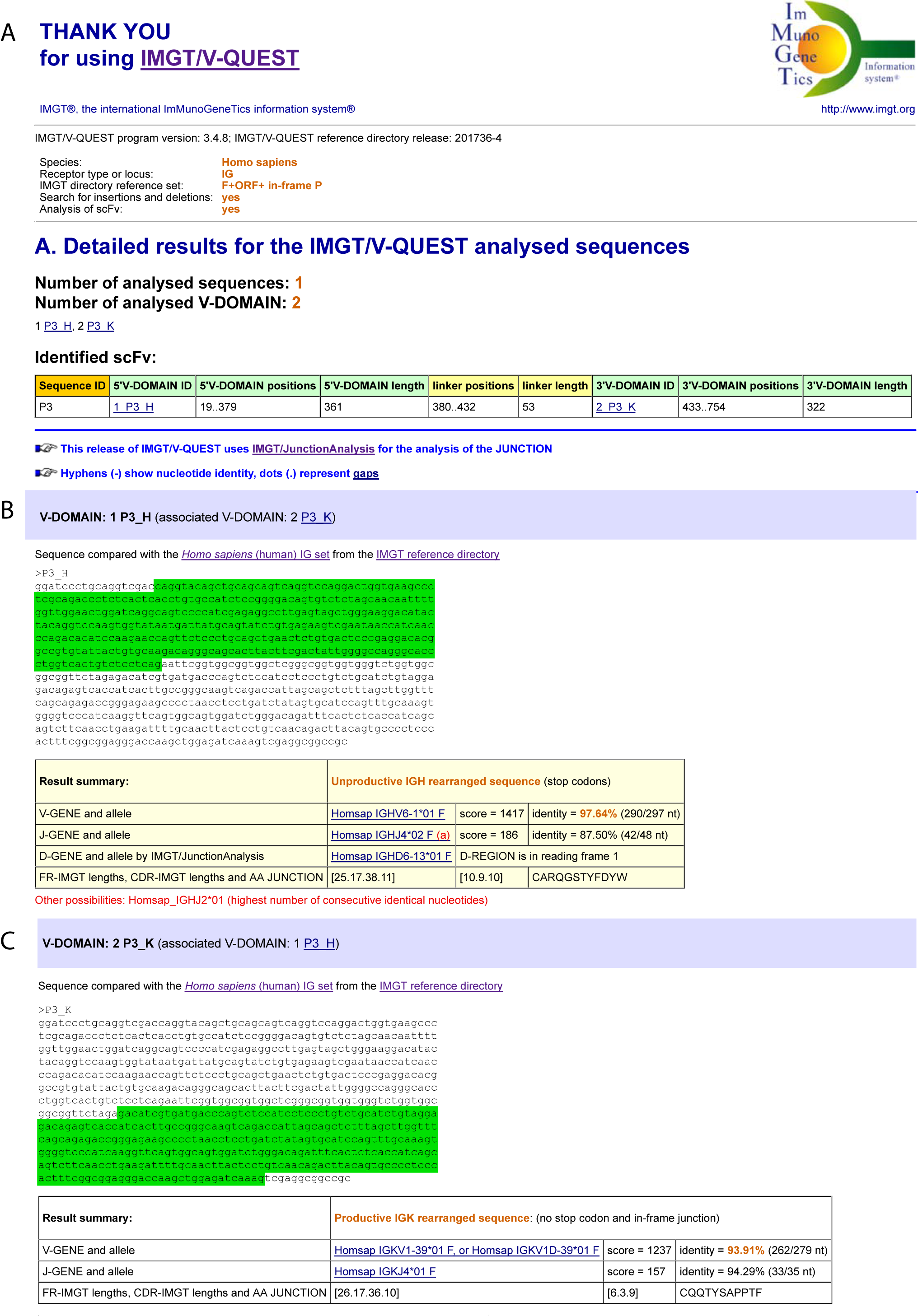

Supplement: Supplementary file 3 [file figure_s3.tif]
