# Supplementary material for: Pacific Biosciences Sequencing and IMGT/HighV-QUEST Analysis of Full-Length Single Chain Fragment Variable from an In Vivo Selected Phage-Display Combinatorial Library
Source: Front Immunol. 2017 Dec 20;8:1796. doi: 10.3389/fimmu.2017.01796 (PMC5742356; doi:10.3389/fimmu.2017.01796)
Supplement: Supplementary file 4 [file figure_s4.pdf]

```
        -----Primer FWD--------|
1       tgcaaattctatttcaaggagac agtcataatgaaatacctattgcctacggcagccgct 60
84      tgcaaattctatttcaaggagac agtcataatgaaatacctattgcctacggcagccgct
9       tgcaaattctatttcaaggagac agtcataatgaaatacctattgcctacggcagccgct
85      tgcaaattctatttcaaggagac agtcataatgaaatacctattgcctacggcagccgct
40      tgcaaattctatttcaaggagac agtcataatgaaatacctattgcctacggcagccgct
7       tgcaaattctatttcaaggagac agtcataatgaaatacctattgcctacggcagccgct
18      tgcaaattctatttcaaggagac agtcataatgaaatacctattgcctacggcagccgct
43      tgcaaattctatttcaaggagac agtcataatgaaatacctattgcctacggcagccgct
58      tgcaaattctatttcaaggagac agtcataatgaaatacctattgcctacggcagccgct
67      tgcaaattctatttcaaggagac agtcataatgaaatacctattgcctacggcagccgct
69      tgcaaattctatttcaaggagac agtcataatgaaatacctattgcctacggcagccgct
79      tgcaaattctatttcaaggagac agtcataatgaaatacctattgcctacggcagccgct
61      tgcaaattctatttcaaggagac agtcataatgaaatacctattgcctacggcagccgct
74      tgcaaattctatttcaaggagac agtcataatgaaatacctattgcctacggcagccgct
81      t-caaattctatttcaaggagac agtcataatgaaatacctattgcctacggcagccgct
5       tgcaaattctatttcaaggagac agtcataatgaaatacctattgcctacggcagccgct
14      tgcaaattctatttcaaggagac agtcataatgaaatacctattgcctacggcagccgct
31      tgcaaattctatttcaaggagac agtcataatgaaatacctattgcctacggcagccgct
34      tgcaaattctatttcaaggagac agtcataatgaaatacctattgcctacggcagccgct
42      tgcaaattctatttcaaggagac agtcataatgaaatacctattgcctacggcagccgct
82      tgcaaattctatttcaaggagac agtcataatgaaatacctattgcctacggcagccgct
22      tgcaaattctatttcaaggagac agtcataatgaaatacctattgcctacggcagccgct
55      tgcaaattctatttcaaggagac agtcataatgaaatacctattgcctacggcagccgct
62      tgcaaattctatttcaaggagac agtcataatgaaatacctattgcctacggcagccgct
77      tgcaaattctatttcaaggagac agtcataatgaaatacctattgcctacggcagccgct
78      tgcaaattctatttcaaggagac agtcataatgaaatacctattgcctacggcagccgct
        * ********************* *************************************

                                                              |<-----
1       ggattgttattactcgcggcccagccggccatggcgggatccctgcaggtcgac caggta 120
84      ggattgttattactcgcggcccagccggccatggcgggatccctgcaggtcgac caggta
9       ggattgttattactcgcggcccagccggccatggcgggatccctgcaggtcgac caggta
85      ggattgttattactcgcggcccagccggccatggcgggatccctgcaggtcgac caggta
40      ggattgttattactcgcggcccagccggccatggcgggatccctgcaggtcgac caggta
7       ggattgttattactcgcggcccagccggccatggcgggatccctgcaggtcgac caggta
18      ggattgttattactcgcggcccagccggccatggcgggatccctgcaggtcgac caggta
43      ggattgttattactcgcggcccagccggccatggcgggatccctgcaggtcgac caggta
58      ggattgttattactcgcggcccagccggccatggcgggatccctgcaggtcgac caggta
67      ggattgttattactcgcggcccagccggccatggcgggatccctgcaggtcgac caggta
69      ggattgttattactcgcggcccagccggccatggcgggatccctgcaggtcgac caggta
79      ggattgttattactcgcggcccagccggccatggcgggatccctgcaggtcgac caggta
61      ggattgttattactcgcggcccagccggccatggcgggatccctgcaggtcgac caggta
74      ggattgttattactcgcggcccagccggccatggcgggatccctgcaggtcgac caggta
81      ggattgttattactcgcggcccagccggccatggcgggatccctgcaggtcgac caggta
5       ggattgttattactcgcggcccagccggccatggcgggatccctgcaggtcgac caggta
14      ggattgttattactcgcggcccagccggccatggcgggatccctgcaggtcgac caggta
31      ggattgttattactcgcggcccagccggccatggcgggatccctgcaggtcgac caggta
34      ggattgttattactcgcggcccagccggccatggcgggatccctgcaggtcgac caggta
42      ggattgttattactcgcggcccagccggccatggcgggatccctgcaggtcgac caggta
82      ggattgttattactcgcggcccagccggccatggcgggatccctgcaggtcgac caggta
22      ggattgttattactcgcggcccagccggccatggcgggatccctgcaggtcgac caggta
55      ggattgttattactcgcggcccagccggccatggcgggatccctgcaggtcgac caggta
62      ggattgttattactcgcggcccagccggccatggcgggatccctgcaggtcgac caggta
77      ggattgttattactcgcggcccagccggccatggcgggatccctgcaggtcgac caggta
78      ggattgttattactcgcggcccagccggccatggcgggatccctgcaggtcgac caggta
        ****************************************************** ******

        ------------------------------------------------------------
1       cagctgcagcagtcaggtccaggactggtgaagccctcgcagaccctctcactcacctgt 180
84      cagctgcagcagtcaggtccaggactggtgaagccctcgcagaccctctcactcacctgt
9       cagctgcagcagtcaggtccaggactggtgaagccctcgcagaccctctcactcacctgt
85      cagctgcagcagtcaggtccaggactggtgaagccctcgcagaccctctcactcacctgt
40      cagctgcagcagtcaggtccaggactggtgaagccctcgcagaccctctcactcacctgt
7       cagctgcagcagtcaggtccaggactggtgaagccctcgcagaccctctcactcacctgt
```

```
18      cagctgcagcagtcaggtccaggactggtgaagccctcgcagaccctctcactcacctgt
43      cagctgcagcagtcaggtccaggactggtgaagccctcgcagaccctctcactcacctgt
58      cagctgcagcagtcaggtccaggactggtgaagccctcgcagaccctctcactcacctgt
67      cagctgcagcagtcaggtccaggactggtgaagccctcgcagaccctctcactcacctgt
69      cagctgcagcagtcaggtccaggactggtgaagccctcgcagaccctctcactcacctgt
79      cagctgcagcagtcaggtccaggactggtgaagccctcgcagaccctctcactcacctgt
61      cagctgcagcagtcaggtccaggactggtgaagccctcgcagaccctctcactcacctgt
74      cagctgcagcagtcaggtccaggactggtgaagccctcgcagaccctctcactcacctgt
81      cagctgcagcagtcaggtccaggactggtgaagccctcgcagaccctctcactcacctgt
5       cagctgcagcagtcaggtccaggactggtgaagccctcgcagaccctctcactcacctgt
14      cagctgcagcagtcaggtccaggactggtgaagccctcgcagaccctctcactcacctgt
31      cagctgcagcagtcaggtccaggactggtgaagccctcgcagaccctctcactcacctgt
34      cagctgcagcagtcaggtccaggactggtgaagccctcgcagaccctctcactcacctgt
42      cagctgcagcagtcaggtccaggactggtgaagccctcgcagaccctctcactcacctgt
82      cagctgcagcagtcaggtccaggactggtgaagccctcgcagaccctctcactcacctgt
22      cagctgcagcagtcaggtccaggactggtgaagccctcgcagaccctctcactcacctgt
55      cagctgcagcagtcaggtccaggactggtgaagccctcgcagaccctctcactcacctgt
62      cagctgcagcagtcaggtccaggactggtgaagccctcgcagaccctctcactcacctgt
77      cagctgcagcagtcaggtccaggactggtgaagccctcgcagaccctctcactcacctgt
78      cagctgcagcagtcaggtccaggactggtgaagccctcgcagaccctctcactcacctgt
        ************************************************************

        ------------------------------------------------------------
1       gccatctccggggacagtgtctctagcaa-caattttggttggaactggatcaggcagtc 239
84      gccatctccggggacagtgtctctagcaaacaattttggttggaactggatcaggcagtc
9       gccatctccggggacagtgtctctagcaa-caattttggttggaactggatcaggcagtc
85      gccatctccggggacagtgtctctagcaa-caattttggttggaactggatcaggcagtc
40      gccatctccggggacagtgtctctagcaa-caattttggttggaactggatcaggcagtc
7       gccatctccggggacagtgtctctagcaa-caattttggttggaactggatcaggcagtc
18      gccatctccggggacagtgtctctagcaa-caattttggttggaactggatcaggcagtc
43      gccatctccggggacagtgtctctagcaa-caattttggttggaactggatcaggcagtc
58      gccatctccggggacagtgtctctagcaa-caattttggttggaactggatcaggcagtc
67      gccatctccggggacagtgtctctagcaa-caattttggttggaactggatcaggcagtc
69      gccatctccggggacagtgtctctagcaa-caattttggttggaactggatcaggcagtc
79      gccatctccggggacagtgtctctagcaa-caattttggttggaactggatcaggcagtc
61      gccatctccggggacagtgtctctagcaa-caattttggttggaactggatcaggcagtc
74      gccatctccggggacagtgtctctagcaa-caattttggttggaactggatcaggcagtc
81      gccatctccggggacagtgtctctagcaa-caattttggttggaactggatcaggcagtc
5       gccatctccggggacagtgtctctagcaa-caattttggttggaactggatcaggcagtc
14      gccatctccggggacagtgtctctagcaa-caattttggttggaactggatcaggcagtc
31      gccatctccggggacagtgtctctagcaa-caattttggttggaactggatcaggcagtc
34      gccatctccggggacagtgtctctagcaa-caattttggttggaactggatcaggcagtc
42      gccatctccggggacagtgtctctagcaa-caattttggttggaactggatcaggcagtc
82      gccatctccggggacagtgtctctagcaa-caattttggttggaactggatcaggcagtc
22      gccatctccggggacagtgtctctagcaa-caattttggttggaactggatcaggcagtc
55      gccatctccggggacagtgtctctagcaa-caattttggttggaactggatcaggcagtc
62      gccatctccggggacagtgtctctagcaa-caattttggttggaactggatcaggcagtc
77      gccatctccggggacagtgtctctagcaa-caattttggttggaactggatcaggcagtc
78      gccatctccggggacagtgtctctagcaa-caattttggttggaactggatcaggcagtc
        ***************************** ******************************

        ------------------------------------------------------------
1       cccatcgagaggccttgagtagctgggaaggacatactacaggtccaagtggtataatga 299
84      cccatcgagaggccttgagtagctgggaaggacatactacaggtccaagtggtataatga
9       cccatcgagaggccttgagtagctgggaaggacatactacaggtccaagtggtataatga
85      cc-atcgagaggccttgagtagctgggaaggacatactacaggtccaagtggtataatga
40      cccatcgagaggccttgagtagctgggaaggacatactacaggtccaagtggtataatga
7       cccatcgagaggccttgagtagctgggaaggacatactacaggtccaagtggtataatga
18      cccatcgagaggccttgagtagctgggaaggacatactacaggtccaagtggtataatga
43      cccatcgagaggccttgagtagctgggaaggacatactacaggtccaagtggtataatga
58      cccatcgagaggccttgagtagctgggaaggacatactacaggtccaagtggtataatga
67      cccatcgagaggccttgagtagctgggaaggacatactacaggtccaagtggtataatga
69      cccatcgagaggccttgagtagctgggaaggacatactacaggtccaagtggtataatga
79      cccatcgagaggccttgagtagctgggaaggacatactacaggtccaagtggtataatga
61      cccatcgagaggccttgagtagctgggaaggacatactacaggtccaagtggtataatga
74      cccatcgagaggccttgagtagctgggaaggacatactacaggtccaagtggtataatga
81      cccatcgagaggccttgagtagctgggaaggacatactacaggtccaagtggtataatga
```

```
5        cccatcgagaggccttgagtagctgggaaggacatactacaggtccaagtggtataatga
14       cccatcgagaggccttgagtagctgggaaggacatactacaggtccaagtggtataatga
31       cccatcgagaggccttgagtagctgggaaggacatactacaggtccaagtggtataatga
34       cccatcgagaggccttgagtagctgggaaggacatactacaggtccaagtggtataatga
42       cccatcgagaggccttgagtagctgggaaggacatactacaggtccaagtggtataatga
82       cccatcgagaggccttgagtagctgggaaggacatactacaggtccaagtggtataatga
22       cccatcgagaggccttgagtagctgggaaggacatactacaggtccaagtggtataatga
55       cccatcgagaggccttgagtagctgggaaggacatactacaggtccaagtggtataatga
62       cccatcgagaggccttgagtagctgggaaggacatactacaggtccaagtggtataatga
77       cccatcgagaggccttgagtagctgggaaggacatactacaggtccaagtggtataatga
78       cccatcgagaggccttgagtagctgggaaggacatactacaggtccaagtggtataatga
         ** *********************************************************

         ------------------------- VH -------------------------------
1        ttatgcagtatctgtgagaagtcgaataaccatcaacccagacacatccaagaaccagtt 359
84       ttatgcagtatctgtgagaagtcgaataaccatcaacccagacacatccaagaaccagtt
9        ttatgcagtatctgtgagaagttgaataaccatcaacccagacacatccaagaaccagtt
85       ttatgcagtatctgtgagaagtcgaataaccatcaacccagacacatccaagaaccagtt
40       ttatgcagtatctgtgagaagtcgaataaccatcaacccagacacatccaagaaccagtt
7        ttatgcagtatctgtgagaagtcgaataaccatcaacccagacacatccaagaaccagtt
18       ttatgcagtatctgtgagaagtcgaataaccatcaacccagacacatccaagaaccagtt
43       ttatgcagtatctgtgagaagtcgaataaccatcaacccagacacatccaagaaccagtt
58       ttatgcagtatctgtgagaagtcgaataaccatcaacccagacacatccaagaaccagtt
67       ttatgcagtatctgtgagaagtcgaataaccatcaacccagacacatccaagaaccagtt
69       ttatgcagtatctgtgagaagtcgaataaccatcaacccagacacatccaagaaccagtt
79       ttatgcagtatctgtgagaagtcgaataaccatcaacccagacacatccaagaaccagtt
61       ttatgcagtatctgtgagaagtcgaataaccatcaacccagacacatccaagaaccagtt
74       ttatgcagtatctgtgagaagtcgaataaccatcaacccagacacatccaagaaccagtt
81       ttatgcagtatctgtgagaagtcgaataaccatcaacccagacacatccaagaaccagtt
5        ttatgcagtatctgtgagaagtcgaataaccatcaacccagacacatccaagaaccagtt
14       ttatgcagtatctgtgagaagtcgaataaccatcaacccagacacatccaagaaccagtt
31       ttatgcagtatctgtgagaagtcgaataaccatcaacccagacacatccaagaaccagtt
34       ttatgcagtatctgtgagaagtcgaataaccatcaacccagacacatccaagaaccagtt
42       ttatgcagtatctgtgagaagtcgaataaccatcaacccagacacatccaagaaccagtt
82       ttatgcagtatctgtgagaagtcgaataaccatcaacccagacacatccaagaaccagtt
22       ttatgcagtatctgtgagaagtcgaataaccatcaacccagacacatccaagaaccagtt
55       ttatgcagtatctgtgagaagtcgaataaccatcaacccagacacatccaagaaccagtt
62       ttatgcagtatctgtgagaagtcgaataaccatcaacccagacacatccaagaaccagtt
77       ttatgcagtatctgtgagaagtcgaataaccatcaacccagacacatccaagaaccagtt
78       ttatgcagtatctgtgagaagtcgaataaccatcaacccagacacatccaagaaccagtt
         ********************** *************************************

         ------------------------------------------------------------
1        ctccctgcagctgaactctgtgactcccgaggacacggccgtgtattactgtgcaagaca 419
84       ctccctgcagctgaactctgtgactcccgaggacacggccgtgtattactgtgcaagaca
9        ctccctgcagctgaactctgtgactcccgaggacacggccgtgtattactgtgcaagaca
85       ctccctgcagctgaactctgtgactcccgaggacacggccgtgtattactgtgcaagaca
40       ctccctgcagctgaactctgtgactcccgaggacacggccgtgtattactgtgcaagaca
7        ctccctgcagctgaactctgtgactcccgaggacacggccgtgtattactgtgcaagaca
18       ctccctgcagctgaactctgtgactcccgaggacacggccgtgtattactgtgcaagaca
43       ctccctgcagctgaactctgtgactcccgaggacacggccgtgtattactgtgcaagaca
58       ctccctgcagctgaactctgtgactcccgaggacacggccgtgtattactgtgcaagaca
67       ctccctgcagctgaactctgtgactcccgaggacacggccgtgtattactgtgcaagaca
69       ctccctgcagctgaactctgtgactcccgaggacacggccgtgtattactgtgcaagaca
79       ctccctgcagctgaactctgtgactcccgaggacacggccgtgtattactgtgcaagaca
61       ctccctgcagctgaactctgtgactcccgaggacacggccgtgtattactgtgcaagaca
74       ctccctgcagctgaactctgtgactcccgaggacacggccgtgtattactgtgcaagaca
81       ctccctgcagctgaactctgtgactcccgaggacacggccgtgtattactgtgcaagaca
5        ctccctgcagctgaactctgtgactcccgaggacacggccgtgtattactgtgcaagaca
14       ctccctgcagctgaactctgtgactcccgaggacacggccgtgtattactgtgcaagaca
31       ctccctgcagctgaactctgtgactcccgaggacacggccgtgtattactgtgcaagaca
34       ctccctgcagctgaactctgtgactcccgaggacacggccgtgtattactgtgcaagaca
42       ctccctgcagctgaactctgtgactcccgaggacacggccgtgtattactgtgcaagaca
82       ctccctgcagctgaactctgtgactcccgaggacacggccgtgtattactgtgcaagaca
22       ctccctgcagctgaactctgtgactcccgaggacacggccgtgtattactgtgcaagaca
55       ctccctgcagctgaactctgtgactcccgaggacacggccgtgtattactgtgcaagaca
62       ctccctgcagctgaactctgtgactcccgaggacacggccgtgtattactgtgcaagaca
```

```
77      ctccctgcagctgaactctgtgactcccgaggacacggccgtgtattactgtgcaagaca
78      ctccctgcagctgaactctgtgactcccgaggacacggccgtgtattactgtgcaagaca
        ************************************************************

        -------------------------------------------------------->|<---
1       gggcagcacttacttcgactattggggccagggcaccctggtcactgtctcctcag aatt 479
84      gggcagcacttacttcgactattggggccagggcaccctggtcactgtctcctcag aatt
9       gggcagcacttacttcgactattggggccagggcaccctggtcactgtctcctcag aatt
85      gggcagcacttacttcgactattggggccagggcaccctggtcactgtctcctcag aatt
40      gggcagcacttacttcgactattggggccagggcaccctggtcactgtctcctcag aatt
7       gggcagcacttacttcgactattggggccagggcaccctggtcactgtctcctcag aatt
18      gggcagcacttacttcgactattggggccagggcaccctggtcactgtctcctcag aatt
43      gggcagcacttacttcgactattggggccagggcaccctggtcactgtctcctcag aatt
58      gggcagcacttacttcgactattggggccagggcaccctggtcactgtctcctcag aatt
67      gggcagcacttacttcgactattggggccagggcaccctggtcactgtctcctcag aatt
69      gggcagcacttacttcgactattggggccagggcaccctggtcactgtctcctcag aatt
79      gggcagcacttacttcgactattggggccagggcaccctggtcactgtctcctcag aatt
61      gggcagcacttacttcgactattggggccagggcaccctggtcactgtctcctcag aatt
74      gggcagcacttacttcgactattggggccagggcaccctggtcactgtctcctcag aatt
81      gggcagcacttacttcgactattggggccagggcaccctggtcactgtctcctcag aatt
5       gggcagcacttacttcgactattggggccagggcaccctggtcactgtctcctcag aatt
14      gggcagcacttacttcgactattggggccagggcaccctggtcactgtctcctcag aatt
31      gggcagcacttacttcgactattggggccagggcaccctggtcactgtctcctcag aatt
34      gggcagcacttacttcgactattggggccagggcaccctggtcactgtctcctcag aatt
42      gggcagcacttacttcgactattggggccagggcaccctggtcactgtctcctcag aatt
82      gggcagcacttacttcgactattggggccagggcaccctggtcactgtctcctcag aatt
22      gggcagcacttacttcgactattggggccagggcaccctggtcactgtctcctcag aatt
55      gggcagcacttacttcgactattggggccagggcaccctggtcactgtctcctcag aatt
62      gggcagcacttacttcgactattggggccagggcaccctggtcactgtctcctcag aatt
77      gggcagcacttacttcgactattggggccagggcaccctggtcactgtctcctcag aatt
78      gggcagcacttacttcgactattggggccagggcaccctggtcactgtctcctcag aatt
        ************************************************************

        --------------- Linker ------------------------->|<----------

1       cggtggcggtggctcgggcggtggtgggtctggtggcggcggttctaga gacatcgtgat 539
84      cggtggcggtggctcgggcggtggtgggtctggtggcggcggttctaga gacatcgtgat
9       cggtggcggtggctcgggcggtggtgggtctggtggcggcggttctaga gacatcgtgat
85      cggtggcggtggctcgggcggtggtgggtctggtggcggcggttctaga gacatcgtgat
40      cggtggcggtggctcgggcggtggtgggtctggtggcggcggttctaga gacatcgtgat
7       cggtggcggtggctcgggcggtggtgggtctggtggcggcggttctaga gacatcgtgat
18      cggtggcggtggctcgggcggtggtgggtctggtggcggcggttctaga gacatcgtgat
43      cggtggcggtggctcgggcggtggtgggtctggtggcggcggttctaga gacatcgtgat
58      cggtggcggtggctcgggcggtggtgggtctggtggcggcggttctaga gacatcgtgat
67      cggtggcggtggctcgggcggtggtgggtctggtggcggcggttctaga gacatcgtgat
69      cggtggcggtggctcgggcggtggtgggtctggtggcggcggttctaga gacatcgtgat
79      cggtggcggtggctcgggcggtggtgggtctggtggcggcggttctaga gacatcgtgat
61      cggtggcggtggctcgggcggtggtgggtctggtggcggcggttctaga gacatcgtgat
74      cggtggcggtggctcgggcggtggtgggtctggtggcggcggttctaga gacatcgtgat
81      cggtggcggtggctcgggcggtggtgggtctggtggcggcggttctaga gacatcgtgat
5       cggtggcggtggctcgggcggtggtgggtctggtggcggcggttctaga gacatcgtgat
14      cggtggcggtggctcgggcggtggtgggtctggtggcggcggttctaga gacatcgtgat
31      cggtggcggtggctcgggcggtggtgggtctggtggcggcggttctaga gacatcgtgat
34      cggtggcggtggctcgggcggtggtgggtctggtggcggcggttctaga gacatcgtgat
42      cggtggcggtggctcgggcggtggtgggtctggtggcggcggttctaga gacatcgtgat
82      cggtggcggtggctcgggcggtggtgggtctggtggcggcggttctaga gacatcgtgat
22      cggtggcggtggctcgggcggtggtgggtctggtggcggcggttctaga gacatcgtgat
55      cggtggcggtggctcgggcggtggtgggtctggtggcggcggttctaga gacatcgtgat
62      cggtggcggtggctcgggcggtggtgggtctggtggcggcggttctaga gacatcgtgat
77      cggtggcggtggctcaggcggtggtgggtctggtggcggcggttctaga gacatcgtgat
78      cggtggcggtggctcgggcggtggtgggtctggtggcggcggttctaga gacatcgtgat
        *************** ********************************* ***********

        ------------------------------------------------------------
1       gacccagtctccatcctccctgtctgcatctgtaggagacagagtcaccatcacttgccg 599
84      gacccagtctccatcctccctgtctgcatctgtaggagacagagtcaccatcacttgccg
9       gacccagtctccatcctccctgtctgcatctgtaggagacagagtcaccatcacttgccg
```

```
85       gacccagtctccatcctccctgtctgcatctgtaggagacagagtcaccatcacttgccg
40       gacccagtctccatcctccctgtctgcatctgtaggagacagagtcaccatcacttgccg
7        gacccggtctccatcctccctgtctgcatctgtaggagacagagtcaccatcacttgccg
18       gacccggtctccatcctccctgtctgcatctgtaggagacagagtcaccatcacttgccg
43       gacccggtctccatcctccctgtctgcatctgtaggagacagagtcaccatcacttgccg
58       gacccggtctccatcctccctgtctgcatctgtaggagacagagtcaccatcacttgccg
67       gacccggtctccatcctccctgtctgcatctgtaggagacagagtcaccatcacttgccg
69       gacccggtctccatcctccctgtctgcatctgtaggagacagagtcaccatcacttgccg
79       gacccggtctccatcctccctgtctgcatctgtaggagacagagtcaccatcacttgccg
61       gacccagtctccatcctccctgtctgcatctgtaggagacagagtcaccatcacttgccg
74       gacccagtctccatcctccctgtctgcatctgtaggagacagagtcaccatcacttgccg
81       gacccagtctccatcctccctgtctgcatctgtaggagacagagtcaccatcacttgccg
5        gacccagtctccatcctccctgtctgcatctgtaggagacagagtcaccatcacttgccg
14       gacccagtctccatcctccctgtctgcatctgtaggagacagagtcaccatcacttgccg
31       gacccagtctccatcctccctgtctgcatctgtaggagacagagtcaccatcacttgccg
34       gacccagtctccatcctccctgtctgcatctgtaggagacagagtcaccatcacttgccg
42       gacccagtctccatcctccctgtctgcatctgtaggagacagagtcaccatcacttgccg
82       gacccagtctccatcctccctgtctgcatctgtaggagacagagtcaccatcacttgccg
22       gacccagtctccatcctccctgtctgcatctgtaggagacagagtcaccatcacttgccg
55       gacccagtctccatcctccctgtctgcatctgtaggagacagagtcaccatcacttgccg
62       gacccagtctccatcctccctgtctgcatctgtaggagacagagtcaccatcacttgccg
77       gacccagtctccatcctccctgtctgcatctgtaggagacagagtcaccatcacttgccg
78       gacccagtctccatcctccctgtctgcatctgtaggagacagagtcaccatcacttgcct
         ***** *****************************************************

         ------------------------------------------------------------
1        ggcaagtcagaccattagcagctctttagcttggtttcagcagagaccgggagaagccc- 658
84       ggcaagtcagaccattagcagctctttagcttggtttcagcagagaccgggagaagccc-
9        ggcaagtcagaccattagcagctctttagcttggtttcagcagagaccgggagaagcccc
85       ggcaagtcagaccattagcagctctttagcttggtttcagcagagaccgggagaagccc-
40       -gcaagtcagaccattagcagctctttagcttggtttcagcagagaccgggagaagccc-
7        ggcaagtcagaccattagcagctctttagcttggtttcagcagagaccgggagaagccc-
18       ggcaagtcagaccattagcagctctttagcttggtttcagcagagaccgggagaagccc-
43       ggcaagtcagaccattagcagctctttagcttggtttcagcagagaccgggagaagccc-
58       ggcaagtcagaccattagcagctctttagcttggtttcagcagagaccgggagaagccc-
67       ggcaagtcagaccattagcagctctttagcttggtttcagcagagaccgggagaagccc-
69       ggcaagtcagaccattagcagctctttagcttggtttcagcagagaccgggagaagccc-
79       ggcaagtcagaccattagcagctctttagcttggtttcagcagagaccgggagaagccc-
61       ggcaagtcagaccattagcagctctttagcttggtttcagcagagaccgggagaagccc-
74       ggcaagtcagaccattagcagctctttagcttggtttcagcagagaccgggagaagccc-
81       ggcaagtcagaccattagcagctctttagcttggtttcagcagagaccgggagaagccc-
5        ggcaagtcagaccattagcagctctttagcttggtttcagcagagaccgggagaagccc-
14       ggcaagtcagaccattagcagctctttagcttggtttcagcagagaccgggagaagccc-
31       ggcaagtcagaccattagcagctctttagcttggtttcagcagagaccgggagaagccc-
34       ggcaagtcagaccattagcagctctttagcttggtttcagcagagaccgggagaagccc-
42       ggcaagtcagaccattagcagctctttagcttggtttcagcagagaccgggagaagccc-
82       ggcaagtcagaccattagcagctctttagcttggtttcagcagagaccgggagaagccc-
22       ggcaagtcagaccattagcagctccttagcttggtttcagcagagaccgggagaagccc-
55       ggcaagtcagaccattagcagctctttggcttggtttcagcagagaccgggagaagccc-
62       ggcaagtcagaccattagcagctctttagcttggtttcagcagagaccgggagaagccc-
77       ggcaagtcagaccattagcagctctttagcttggtttcagcagagaccgggagaagccc-
78       ggcaagtcagaccattagcagctctttagcttggtttcagcagagaccgggagaagccc-
          *********************** ** ******************************

         ----------------------- VL ---------------------------------
1        -ctaacctcctgatctatagtgcatccagtttgcaaagtggggtcccatcaaggttcagt 717
84       -ctaacctcctgatctatagtgcatccagtttgcaaagtggggtcccatcaaggttcagt
9        ctaaacctcctgatctatagtgcatccagtttgcaaagtggggtcccatcaaggttcagt
85       -ctaacctcctgatctatagtgcatccagtttgcaaagtggggtcccatcaaggttcagt
40       -ctaacctcctgatctatagtgcatccagtttgcaaagtggggtcccatcaaggttcagt
7        -ctaacctcctgatctatagtgcatccaatttgcaaagtggggtcccatcaaggttcagt
18       -ctaacctcctgatctatagtgcatccaatttgcaaagtggggtcccatcaaggttcagt
43       -ctaacctcctgatctatagtgcatccaatttgcaaagtggggtcccatcaaggttcagt
58       -ctaacctcctgatctatagtgcatccaatttgcaaagtggggtcccatcaaggttcagt
67       -ctaacctcctgatctatagtgcatccaatttgcaaagtggggtcccatcaaggttcagt
69       -ctaacctcctgatctatagtgcatccaatttgcaaagtggggtcccatcaaggttcagt
79       -ctaacctcctgatctatagtgcatccaatttgcaaagtggggtcccatcaaggttcagt
```

```
61        -ctaacctcctgatctatagtgcatccagtttgcaaagtggggtcccatcaaggttcagt
74        -ctaacctcctgatctatagtgcatccagtttgcaaagtggggtcccatcaaggttcagt
81        -ctaacctcctgatctatagtgcatccagtttgcaaagtggggtcccatcaaggttcggt
5         -ctaacctcctgatctatagtgcatccagtttgcaaagtggggtcccatcaaggttcagt
14        -ctaacctcctgatctatagtgcatccagtttgcaaagtggggtcccatcaaggttcagt
31        -ctaacctcctgatctatagtgcatccagtttgcaaagtggggtcccatcaaggttcagt
34        -ctaacctcctgatctatagtgcatccagtttgcaaagtggggtcccatcaaggttcagt
42        -ctaacctcctgatctatagtgcatccagtttgcaaagtggggtcccatcaaggttcagt
82        -ctaacctcctgatctatagtgcatccagtttgcaaagtggggtcccatcaaggttcagt
22        -ctaacctcctgatctatagtgcatccagtttgcaaagtggggtcccatcaaggttcagt
55        -ctaacctcctgatctatagtgcatccagtttgcaaagtggggtcccatcaaggttcagt
62        -ctaacctcctgatctatagtgcatccagtttgcaaagtggggtcccatcaaggttcagt
77        -ctaacctcctgatctatagtgcatccagtttgcaaagtggggtcccatcaaggttcagt
78        -ctaacctcctgatctatagtgcatccagtttgcaaagtggggtcccatcaaggttcagt
             ************************* **************************** **

          ------------------------------------------------------------
1         ggcagtggatctgggacagatttcactctcaccatcagcagtctt-caacctgaagattt 776
84        ggcagtggatctgggacagatttcactctcaccatcagcagtctttcaacctgaagattt
9         ggcagtggatctgggacagatttcactctcaccatcagcagtctt-caacctgaagattt
85        ggcagtggatctgggacagatttcactctcaccatcagcagtctt-caacctgaagattt
40        ggcagtggatctgggacagatttcactctcaccatcagcagtctt-caacctgaagattt
7         ggcagtggatctgggacagatttcactctcaccatcagcggtctt-caacctgaagattt
18        ggcagtggatctgggacagatttcactctcaccatcagcggtctt-caacctgaagattt
43        ggcagtggatctgggacagatttcactctcaccatcagcggtctt-caacctgaagattt
58        ggcagtggatctgggacagatttcactctcaccatcagcggtctt-caacctgaagattt
67        ggcagtggatctgggacagatttcactctcaccatcagcggtctt-caacctgaagattt
69        ggcagtggatctgggacagatttcactctcaccatcagcggtctt-caacctgaagattt
79        ggcagtggatctgggacagatttcactctcaccatcagcggtctt-caacctgaagattt
61        ggcagtggatctgggacagattttactctcaccatcagcagtctt-caacctgaagattt
74        ggcagtggatctgggacagattttactctcaccatcagcagtctt-caacctgaagattt
81        ggcagtggatctgggacagatttcactctcaccatcagcagtctt-caacctgaagattt
5         ggtagtggatctgggacagatttcacctcaccatcagcagtctt-caacctgaagattt
14        ggtagtggatctgggacagatttcacctcaccatcagcagtctt-caacctgaagattt
31        ggtagtggatctgggacagatttcacctcaccatcagcagtctt-caacctgaagattt
34        ggtagtggatctgggacagatttcacctcaccatcagcagtctt-caacctgaagattt
42        ggtagtggatctgggacagatttcacctcaccatcagcagtctt-caacctgaagattt
82        ggtagtggatctgggacagatttcacctcaccatcagcagtctt-caacctgaagattt
22        ggcagtggatctgggacagatttcactctcaccatcagcagtctt-caacctgaagattt
55        ggcagtggatctgggacagatttcactctcaccatcagcagtctt-caacctgaagattt
62        ggcagtggatctgggacaaatttcactctcaccatcagcagtctt-caacctgaagattt
77        ggcagtggatctgggacagatttcactctcaccatcagcagtctt-caacctgaagattt
78        ggcagtggatctgggacagatttcactctcaccatcagcagtctt-caacctgaagattt
          ** *************** **** ** ************ ***** **************

          ------------------------------------------------------------
1         tgcaacttactcctgtcaacagacttacagtgcccctcccactttcggcggagggaccaa 836
84        tgcaacttactcctgtcaacagacttacagtgcccctcccactttcggcggagggaccaa
9         tgcaacttactcctgtcaacagacttacagtgcccctcccactttcggcggagggaccaa
85        tgcaacttactcctgtcaacagacttacagtgcccctcccactttcggcggagggaccaa
40        tgcaacttactcctgtcaacagacttacagtgcccctcccactttcggcggagggaccaa
7         tgcaacttactcctgtcaacagacttacagtgcccctcccactttcggcggagggaccaa
18        tgcaacttactcctgtcaacagacttacagtgcccctcccactttcggcggagggaccaa
43        tgcaacttactcctgtcaacagacttacagtgcccctcccactttcggcggagggaccaa
58        tgcaacttactcctgtcaacagacttacagtgcccctcccactttcggcggagggaccaa
67        tgcaacttactcctgtcaacagacttacagtgcccctcccactttcggcggagggaccaa
69        tgcaacttactcctgtcaacagacttacagtgcccctcccactttcggcggagggaccaa
79        tgcaacttactcctgtcaacagacttacagtgcccctcccactttcggcggagggaccaa
61        tgcaacttactcctgtcaacagacttacagtgcccctcccactttcggcggagggaccaa
74        tgcaacttactcctgtcaacagacttacagtgcccctcccactttcggcggagggaccaa
81        tgcaacttactcctgtcaacagacttacagtgcccctcccactttcggcggagggaccaa
5         tgcaacttactcctgtcaacagacttacagtgcccctcccactttcggcggagggaccaa
14        tgcaacttactcctgtcaacagacttacagtgcccctcccactttcggcggagggaccaa
31        tgcaacttactcctgtcaacagacttacagtgcccctcccactttcggcggagggaccaa
34        tgcaacttactcctgtcaacagacttacagtgcccctcccactttcggcggagggaccaa
42        tgcaacttactcctgtcaacagacttacagtgcccctcccactttcggcggagggaccaa
82        tgcaacttactcctgtcaacagacttacagtgcccctcccactttcggcggagggaccaa
```

```
22        tgcaacttactcctgtcaacagacttacagtgcccctcccactttcggcggagggaccaa
55        tgcaacttactcctgtcaacagacttacagtgcccctcccactttcggcggagggaccaa
62        tgcaacttactcctgtcaacagacttacagtgcccctcccactttcggcggagggaccaa
77        tgcaacttactcctgtcaacagacttacagtgcccctcccactttcggcggagggaccaa
78        tgcaacttactcctgtcaacagacttacagtgcccctcccactttcggcggagggaccaa
          ************************************************************

          ------------->|
1         gctggagatcaaag tcgaggcggccgcatagagaaacaacggcaacgggaacggcacagg 896
84        gctggagatcaaag tcgaggcggccgcatagagaaacaacggcaacgggaacggcacagg
9         gctggagatcaaag tcgaggcggccgcatagagaaacaacggcaacgggaacggcacagg
85        gctggagatcaaag tcgaggcggccgcatagagaaacaacggcaacgggaacggcacagg
40        gctggagatcaaag tcgaggcggccgcatagagaaacaacggcaacgggaacggcacagg
7         ggtggagatcaaag tcgaggcggccgcatagagaaacaacggcaacgggaacggcacagg
18        ggtggagatcaaag tcgaggcggccgcatagagaaacaacggcaacgggaacggcacagg
43        ggtggagatcaaag tcgaggcggccgcatagagaaacaacggcaacgggaacggcacagg
58        ggtggagatcaaag tcgaggcggccgcatagagaaacaacggcaacggg-----------
67        ggtggagatcaaag tcgaggcggccgcatagagaaacaacggcaacgggaacggcacagg
69        ggtggagatcaaag tcgaggcggccgcatagagaaacaacggcaacgggaacggcacagg
79        ggtggagatcaaag tcgaggcggccgcatagagaaacaacggcaacgggaacggcacagg
61        agtggatatcaaag tcgaggcggccgcatagagaaacaacggcaacgggaacggcacagg
74        agtggatatcaaag tcgaggcggccgcatagagaaacaacggcaacgggaacggcacagg
81        gctggagatcaaag tcgaggcggccgcatagagaaacaacggcaacgggaacggcacagg
5         gctggagatcaaag tcgaggcggccgcatagagaaacaacggcaacgggaacggcacagg
14        gctggagatcaaag tcgaggcggccgcatagagaaacaacggcaacgggaacggcacagg
31        gctggagatcaaag tcgaggcggccgcatagagaaacaacggcaacgggaacggcacagg
34        gctggagatcaaag tcgaggcggccgcatagagaaacaacggcaacgggaacggcacagg
42        gctggagatcaaag tcgaggcggccgcatagagaaacaacggcaacgggaacggcacagg
82        gctggagatcaaag tcgaggcggccgcatagagaaacaacggcaacgggaacggcacagg
22        gctggagatcaaag tcgaggcggccgcatagagaaacaacggcaacgggaacggcacagg
55        gctggagatcaaag tcgaggcggccgcatagagaaacaacggcaacgggaacggcacagg
62        gctggagatcaaag tcgaggcggccgcatagagaaacaacggcaacgggaacggcacagg
77        gctggagatcaaag tcgaggcggccgcatagagaaacaacggcaacgggaacggcacagg
78        gctggagatcaaag tcgaggcggccgcatagagaaacaacggcaacgggaacggcacagg
            **** ******* ***********************************


1         agatgttttgcttaacgttctttcgaagtgtgccggctctaaaaaattcaggcctgctcc 956
84        agatgttttgcttaacgttctttcgaagtgtgccggctctaaaaaattcaggcctgctcc
9         agatgttttgcttaacgttctttcgaagtgtgccggctctaaaaaattcaggcctgctcc
85        agatgttttgcttaacgttctttcgaagtgtgccggctctaaaaaattcaggcctgctcc
40        agatgttttgcttaacgttctttcgaagtgtgccggctctaaaaaattcaggcctgctcc
7         agatgttttgcttaacgttctttcgaagtgtgccggctctaaaaaattcaggcctgctcc
18        agatgttttgcttaacgttctttcgaagtgtgccggctctaaaaaattcaggcctgctcc
43        agatgttttgcttaacgttctttcgaagtgtgccggctctaaaaaattcaggcctgctcc
58        ------------------------------------------------------------
67        agatgttttgcttaacgttctttcgaagtgtgccggctctaaaaaattcaggcctgctcc
69        agatgttttgcttaacgttctttcgaagtgtgccggctctaaaaaattcaggcctgctcc
79        agatgttttgcttaacgttctttcgaagtgtgccggctctaaaaaattcaggcctgctcc
61        agatgttttgcttaacgttctttcgaagtgtgccggctctaaaaaattcaggcctgctcc
74        agatgttttgcttaacgttctttcgaagtgtgccggctctaaaaaattcaggcctgctcc
81        agatgttttgcttaacgttctttcgaagtgtgccggctctaaaaaattcaggcctgctcc
5         agatgttttgcttaacgttctttcgaagtgtgccggctctaaaaaattcaggcctgctcc
14        agatgttttgcttaacgttctttcgaagtgtgccggctctaaaaaattcaggcctgctcc
31        agatgttttgcttaacgttctttcgaagtgtgccggctctaaaaaattcaggcctgctcc
34        agatgttttgcttaacgttctttcgaagtgtgccggctctaaaaaattcaggcctgctcc
42        agatgttttgcttaacgttctttcgaagtgtgccggctctaaaaaattcaggcctgctcc
82        agatgttttgcttaacgttctttcgaagtgtgccggctctaaaaaattcaggcctgctcc
22        agatgttttgcttaacgttctttcgaagtgtgccggctctaaaaaattcaggcctgctcc
55        agatgttttgcttaacgttctttcgaagtgtgccggctctaaaaaattcaggcctgctcc
62        agatgttttgcttaacgttctttcgaagtgtgccggctctaaaaaattcaggcctgctcc
77        agatgttttgcttaacgttctttcgaagtgtgccggctctaaaaaattcaggcctgctcc
78        agatgttttgcttaacgttctttcgaagtgtgccggctctaaaaaattcaggcctgctcc

           |<--- Primer REV --->
1         c gccgctgcttttgcacgtga 977
84        c gccgctgcttttgcacgtga
```

```
9        c gccgctgcttttgcacgtga
85       c gccgctgcttttgcacgtga
40       c gccgctgcttttgcacgtga
7        c gccgctgcttttgcacgtga
18       c gccgctgcttttgcacgtga
43       c gccgctgcttttgcacgtga
58       - --------------------
67       c gccgctgcttttgcacgtga
69       c gccgctgcttttgcacgtga
79       c gccgctgcttttgcacgtga
61       c gccgctgcttttgcacgtga
74       c gccgctgcttttgcacgtg-
81       c g-------------------
5        c gccgctgcttttgcacgtga
14       c gccgctgcttttgcacgtga
31       c gccgctgcttttgcacgtga
34       c gccgctgcttttgcacgtga
42       c gccgctgcttttgcacgtga
82       c gccgctgcttttgcacgtga
22       c gccgctgcttttgcacgtga
55       c gccgctgcttttgcacgtga
62       c gccgctgcttttgcacgtga
77       c gccgctgcttttgcacgtga
78       c gccgctgcttttgcacg---
```
