# Supplementary material for: Pacific Biosciences Sequencing and IMGT/HighV-QUEST Analysis of Full-Length Single Chain Fragment Variable from an In Vivo Selected Phage-Display Combinatorial Library
Source: Front Immunol. 2017 Dec 20;8:1796. doi: 10.3389/fimmu.2017.01796 (PMC5742356; doi:10.3389/fimmu.2017.01796)
Supplement: Supplementary file 5 [file figure_s5.pdf]

Sequences aligned with Homsap_IGHV6-1*01

3. V-REGION translation

```
                                    <--------------------------------------------  FR1 - IMGT
                                    1               5                   10                  15
                                     Q   V   Q   L   Q   Q   S   G   P       G   L   V   K   P
J04097 Homsap IGHV6-1*01 F          cag gta cag ctg cag cag tca ggt cca ... gga ctg gtg aag ccc

P3_H                                --- --- --- --- --- --- --- --- --- ... --- --- --- --- ---

7_H                                 --- --- --- --- --- --- --- --- --- ... --- --- --- --- ---

18_H                                --- --- --- --- --- --- --- --- --- ... --- --- --- --- ---

43_H                                --- --- --- --- --- --- --- --- --- ... --- --- --- --- ---

58_H                                --- --- --- --- --- --- --- --- --- ... --- --- --- --- ---

67_H                                --- --- --- --- --- --- --- --- --- ... --- --- --- --- ---

69_H                                --- --- --- --- --- --- --- --- --- ... --- --- --- --- ---

79_H                                --- --- --- --- --- --- --- --- --- ... --- --- --- --- ---

61_H                                --- --- --- --- --- --- --- --- --- ... --- --- --- --- ---

74_H                                --- --- --- --- --- --- --- --- --- ... --- --- --- --- ---

5_H                                 --- --- --- --- --- --- --- --- --- ... --- --- --- --- ---

14_H                                --- --- --- --- --- --- --- --- --- ... --- --- --- --- ---

31_H                                --- --- --- --- --- --- --- --- --- ... --- --- --- --- ---

34_H                                --- --- --- --- --- --- --- --- --- ... --- --- --- --- ---

42_H                                --- --- --- --- --- --- --- --- --- ... --- --- --- --- ---

82_H                                --- --- --- --- --- --- --- --- --- ... --- --- --- --- ---


                                    ------------------------------------------> ________________
                                                    20                  25                  30
                                     S   Q   T   L   S   L   T   C   A   I   S   G   D   S   V
J04097 Homsap IGHV6-1*01 F          tcg cag acc ctc tca ctc acc tgt gcc atc tcc ggg gac agt gtc

P3_H                                --- --- --- --- --- --- --- --- --- --- --- --- --- --- ---

7_H                                 --- --- --- --- --- --- --- --- --- --- --- --- --- --- ---

18_H                                --- --- --- --- --- --- --- --- --- --- --- --- --- --- ---

43_H                                --- --- --- --- --- --- --- --- --- --- --- --- --- --- ---

58_H                                --- --- --- --- --- --- --- --- --- --- --- --- --- --- ---

67_H                                --- --- --- --- --- --- --- --- --- --- --- --- --- --- ---

69_H                                --- --- --- --- --- --- --- --- --- --- --- --- --- --- ---

79_H                                --- --- --- --- --- --- --- --- --- --- --- --- --- --- ---

61_H                                --- --- --- --- --- --- --- --- --- --- --- --- --- --- ---

74_H                                --- --- --- --- --- --- --- --- --- --- --- --- --- --- ---

5_H                                 --- --- --- --- --- --- --- --- --- --- --- --- --- --- ---

14_H                                --- --- --- --- --- --- --- --- --- --- --- --- --- --- ---

31_H                                --- --- --- --- --- --- --- --- --- --- --- --- --- --- ---

34_H                                --- --- --- --- --- --- --- --- --- --- --- --- --- --- ---

42_H                                --- --- --- --- --- --- --- --- --- --- --- --- --- --- ---
```

```
82_H                                --- --- --- --- --- --- --- --- --- --- --- --- --- --- ---

                                    __  CDR1 - IMGT  ______________ <--------------------------
                                                    35                  40                  45
                                     S           S   N   S   A   A   W   N   W   I   R   Q   S
J04097 Homsap IGHV6-1*01 F          tct ... ... agc aac agt gct gct tgg aac tgg atc agg cag tcc
                                                         N   F   G
P3_H                                --- ... ... --- --- -a- tt- -g- --- --- --- --- --- --- ---
                                                         N   F   G
7_H                                 --- ... ... --- --- -a- tt- -g- --- --- --- --- --- --- ---
                                                         N   F   G
18_H                                --- ... ... --- --- -a- tt- -g- --- --- --- --- --- --- ---
                                                         N   F   G
43_H                                --- ... ... --- --- -a- tt- -g- --- --- --- --- --- --- ---
                                                         N   F   G
58_H                                --- ... ... --- --- -a- tt- -g- --- --- --- --- --- --- ---
                                                         N   F   G
67_H                                --- ... ... --- --- -a- tt- -g- --- --- --- --- --- --- ---
                                                         N   F   G
69_H                                --- ... ... --- --- -a- tt- -g- --- --- --- --- --- --- ---
                                                         N   F   G
79_H                                --- ... ... --- --- -a- tt- -g- --- --- --- --- --- --- ---
                                                         N   F   G
61_H                                --- ... ... --- --- -a- tt- -g- --- --- --- --- --- --- ---
                                                         N   F   G
74_H                                --- ... ... --- --- -a- tt- -g- --- --- --- --- --- --- ---
                                                         N   F   G
5_H                                 --- ... ... --- --- -a- tt- -g- --- --- --- --- --- --- ---
                                                         N   F   G
14_H                                --- ... ... --- --- -a- tt- -g- --- --- --- --- --- --- ---
                                                         N   F   G
31_H                                --- ... ... --- --- -a- tt- -g- --- --- --- --- --- --- ---
                                                         N   F   G
34_H                                --- ... ... --- --- -a- tt- -g- --- --- --- --- --- --- ---
                                                         N   F   G
42_H                                --- ... ... --- --- -a- tt- -g- --- --- --- --- --- --- ---
                                                         N   F   G
82_H                                --- ... ... --- --- -a- tt- -g- --- --- --- --- --- --- ---

                                     FR2 - IMGT  -------------------------> ______________  CDR2
                                                    50                  55                  60
                                     P   S   R   G   L   E   W   L   G   R   T   Y   Y   R   S
J04097 Homsap IGHV6-1*01 F          cca tcg aga ggc ctt gag tgg ctg gga agg aca tac tac agg tcc
                                                             *
P3_H                                --- --- --- --- --- --- -a- --- --- --- --- --- --- --- ---
                                                             *
7_H                                 --- --- --- --- --- --- -a- --- --- --- --- --- --- --- ---
                                                             *
18_H                                --- --- --- --- --- --- -a- --- --- --- --- --- --- --- ---
                                                             *
43_H                                --- --- --- --- --- --- -a- --- --- --- --- --- --- --- ---
                                                             *
58_H                                --- --- --- --- --- --- -a- --- --- --- --- --- --- --- ---
                                                             *
67_H                                --- --- --- --- --- --- -a- --- --- --- --- --- --- --- ---
                                                             *
69_H                                --- --- --- --- --- --- -a- --- --- --- --- --- --- --- ---
                                                             *
79_H                                --- --- --- --- --- --- -a- --- --- --- --- --- --- --- ---
                                                             *
61_H                                --- --- --- --- --- --- -a- --- --- --- --- --- --- --- ---
                                                             *
74_H                                --- --- --- --- --- --- -a- --- --- --- --- --- --- --- ---
                                                             *
5_H                                 --- --- --- --- --- --- -a- --- --- --- --- --- --- --- ---
                                                             *
14_H                                --- --- --- --- --- --- -a- --- --- --- --- --- --- --- ---
                                                             *
31_H                                --- --- --- --- --- --- -a- --- --- --- --- --- --- --- ---
                                                             *
34_H                                --- --- --- --- --- --- -a- --- --- --- --- --- --- --- ---
                                                             *
42_H                                --- --- --- --- --- --- -a- --- --- --- --- --- --- --- ---
                                                             *
82_H                                --- --- --- --- --- --- -a- --- --- --- --- --- --- --- ---
```

```
                                     - IMGT  __________ <----------------------------------------
                                                     65                   70                   75
                                        K   W   Y   N   D   Y   A   V   S   V   K       S   R
J04097 Homsap IGHV6-1*01 F           ... aag tgg tat aat gat tat gca gta tct gtg aaa ... agt cga
                                                                            R
P3_H                                 ... --- --- --- --- --- --- --- --- --- --- -g- ... --- ---
                                                                            R
7_H                                  ... --- --- --- --- --- --- --- --- --- --- -g- ... --- ---
                                                                            R
18_H                                 ... --- --- --- --- --- --- --- --- --- --- -g- ... --- ---
                                                                            R
43_H                                 ... --- --- --- --- --- --- --- --- --- --- -g- ... --- ---
                                                                            R
58_H                                 ... --- --- --- --- --- --- --- --- --- --- -g- ... --- ---
                                                                            R
67_H                                 ... --- --- --- --- --- --- --- --- --- --- -g- ... --- ---
                                                                            R
69_H                                 ... --- --- --- --- --- --- --- --- --- --- -g- ... --- ---
                                                                            R
79_H                                 ... --- --- --- --- --- --- --- --- --- --- -g- ... --- ---
                                                                            R
61_H                                 ... --- --- --- --- --- --- --- --- --- --- -g- ... --- ---
                                                                            R
74_H                                 ... --- --- --- --- --- --- --- --- --- --- -g- ... --- ---
                                                                            R
5_H                                  ... --- --- --- --- --- --- --- --- --- --- -g- ... --- ---
                                                                            R
14_H                                 ... --- --- --- --- --- --- --- --- --- --- -g- ... --- ---
                                                                            R
31_H                                 ... --- --- --- --- --- --- --- --- --- --- -g- ... --- ---
                                                                            R
34_H                                 ... --- --- --- --- --- --- --- --- --- --- -g- ... --- ---
                                                                            R
42_H                                 ... --- --- --- --- --- --- --- --- --- --- -g- ... --- ---
                                                                            R
82_H                                 ... --- --- --- --- --- --- --- --- --- --- -g- ... --- ---

                                     ------------------------------  FR3 - IMGT  --------------
                                                     80                   85                   90
                                      I   T   I   N   P   D   T   S   K   N   Q   F   S   L   Q
J04097 Homsap IGHV6-1*01 F           ata acc atc aac cca gac aca tcc aag aac cag ttc tcc ctg cag

P3_H                                 --- --- --- --- --- --- --- --- --- --- --- --- --- --- ---

7_H                                  --- --- --- --- --- --- --- --- --- --- --- --- --- --- ---

18_H                                 --- --- --- --- --- --- --- --- --- --- --- --- --- --- ---

43_H                                 --- --- --- --- --- --- --- --- --- --- --- --- --- --- ---

58_H                                 --- --- --- --- --- --- --- --- --- --- --- --- --- --- ---

67_H                                 --- --- --- --- --- --- --- --- --- --- --- --- --- --- ---

69_H                                 --- --- --- --- --- --- --- --- --- --- --- --- --- --- ---

79_H                                 --- --- --- --- --- --- --- --- --- --- --- --- --- --- ---

61_H                                 --- --- --- --- --- --- --- --- --- --- --- --- --- --- ---

74_H                                 --- --- --- --- --- --- --- --- --- --- --- --- --- --- ---

5_H                                  --- --- --- --- --- --- --- --- --- --- --- --- --- --- ---

14_H                                 --- --- --- --- --- --- --- --- --- --- --- --- --- --- ---

31_H                                 --- --- --- --- --- --- --- --- --- --- --- --- --- --- ---

34_H                                 --- --- --- --- --- --- --- --- --- --- --- --- --- --- ---

42_H                                 --- --- --- --- --- --- --- --- --- --- --- --- --- --- ---

82_H                                 --- --- --- --- --- --- --- --- --- --- --- --- --- --- ---
```

```
                                    ------------------------------------------------------>  CDR
                                                    95                  100             104
                                     L   N   S   V   T   P   E   D   T   A   V   Y   Y   C   A
J04097 Homsap IGHV6-1*01 F          ctg aac tct gtg act ccc gag gac acg gct gtg tat tac tgt gca

P3_H                                --- --- --- --- --- --- --- --- --- --c --- --- --- --- ---

7_H                                 --- --- --- --- --- --- --- --- --- --c --- --- --- --- ---

18_H                                --- --- --- --- --- --- --- --- --- --c --- --- --- --- ---

43_H                                --- --- --- --- --- --- --- --- --- --c --- --- --- --- ---

58_H                                --- --- --- --- --- --- --- --- --- --c --- --- --- --- ---

67_H                                --- --- --- --- --- --- --- --- --- --c --- --- --- --- ---

69_H                                --- --- --- --- --- --- --- --- --- --c --- --- --- --- ---

79_H                                --- --- --- --- --- --- --- --- --- --c --- --- --- --- ---

61_H                                --- --- --- --- --- --- --- --- --- --c --- --- --- --- ---

74_H                                --- --- --- --- --- --- --- --- --- --c --- --- --- --- ---

5_H                                 --- --- --- --- --- --- --- --- --- --c --- --- --- --- ---

14_H                                --- --- --- --- --- --- --- --- --- --c --- --- --- --- ---

31_H                                --- --- --- --- --- --- --- --- --- --c --- --- --- --- ---

34_H                                --- --- --- --- --- --- --- --- --- --c --- --- --- --- ---

42_H                                --- --- --- --- --- --- --- --- --- --c --- --- --- --- ---

82_H                                --- --- --- --- --- --- --- --- --- --c --- --- --- --- ---


                                    3 -  IMGT

                                     R
J04097 Homsap IGHV6-1*01 F          aga ga
                                         Q   G   S   T   Y   F   D   Y   W   G   Q   G   T   L
P3_H                                --- c-g ggc agc act tac ttc gac tat tgg ggc cag ggc acc ctg
                                         Q   G   S   T   Y   F   D   Y   W   G   Q   G   T   L
7_H                                 --- c-g ggc agc act tac ttc gac tat tgg ggc cag ggc acc ctg
                                         Q   G   S   T   Y   F   D   Y   W   G   Q   G   T   L
18_H                                --- c-g ggc agc act tac ttc gac tat tgg ggc cag ggc acc ctg
                                         Q   G   S   T   Y   F   D   Y   W   G   Q   G   T   L
43_H                                --- c-g ggc agc act tac ttc gac tat tgg ggc cag ggc acc ctg
                                         Q   G   S   T   Y   F   D   Y   W   G   Q   G   T   L
58_H                                --- c-g ggc agc act tac ttc gac tat tgg ggc cag ggc acc ctg
                                         Q   G   S   T   Y   F   D   Y   W   G   Q   G   T   L
67_H                                --- c-g ggc agc act tac ttc gac tat tgg ggc cag ggc acc ctg
                                         Q   G   S   T   Y   F   D   Y   W   G   Q   G   T   L
69_H                                --- c-g ggc agc act tac ttc gac tat tgg ggc cag ggc acc ctg
                                         Q   G   S   T   Y   F   D   Y   W   G   Q   G   T   L
79_H                                --- c-g ggc agc act tac ttc gac tat tgg ggc cag ggc acc ctg
                                         Q   G   S   T   Y   F   D   Y   W   G   Q   G   T   L
61_H                                --- c-g ggc agc act tac ttc gac tat tgg ggc cag ggc acc ctg
                                         Q   G   S   T   Y   F   D   Y   W   G   Q   G   T   L
74_H                                --- c-g ggc agc act tac ttc gac tat tgg ggc cag ggc acc ctg
                                         Q   G   S   T   Y   F   D   Y   W   G   Q   G   T   L
5_H                                 --- c-g ggc agc act tac ttc gac tat tgg ggc cag ggc acc ctg
                                         Q   G   S   T   Y   F   D   Y   W   G   Q   G   T   L
14_H                                --- c-g ggc agc act tac ttc gac tat tgg ggc cag ggc acc ctg
                                         Q   G   S   T   Y   F   D   Y   W   G   Q   G   T   L
31_H                                --- c-g ggc agc act tac ttc gac tat tgg ggc cag ggc acc ctg
                                         Q   G   S   T   Y   F   D   Y   W   G   Q   G   T   L
34_H                                --- c-g ggc agc act tac ttc gac tat tgg ggc cag ggc acc ctg
                                         Q   G   S   T   Y   F   D   Y   W   G   Q   G   T   L
42_H                                --- c-g ggc agc act tac ttc gac tat tgg ggc cag ggc acc ctg
                                         Q   G   S   T   Y   F   D   Y   W   G   Q   G   T   L
82_H                                --- c-g ggc agc act tac ttc gac tat tgg ggc cag ggc acc ctg
```

```
J04097 Homsap IGHV6-1*01 F

                                    V   T   V   S   S
P3_H                               gtc act gtc tcc tca g  Homsap IGHJ4*02 F
                                    V   T   V   S   S
7_H                                gtc act gtc tcc tca g  Homsap IGHJ4*02 F
                                    V   T   V   S   S
18_H                               gtc act gtc tcc tca g  Homsap IGHJ4*02 F
                                    V   T   V   S   S
43_H                               gtc act gtc tcc tca g  Homsap IGHJ4*02 F
                                    V   T   V   S   S
58_H                               gtc act gtc tcc tca g  Homsap IGHJ4*02 F
                                    V   T   V   S   S
67_H                               gtc act gtc tcc tca g  Homsap IGHJ4*02 F
                                    V   T   V   S   S
69_H                               gtc act gtc tcc tca g  Homsap IGHJ4*02 F
                                    V   T   V   S   S
79_H                               gtc act gtc tcc tca g  Homsap IGHJ4*02 F
                                    V   T   V   S   S
61_H                               gtc act gtc tcc tca g  Homsap IGHJ4*02 F
                                    V   T   V   S   S
74_H                               gtc act gtc tcc tca g  Homsap IGHJ4*02 F
                                    V   T   V   S   S
5_H                                gtc act gtc tcc tca g  Homsap IGHJ4*02 F
                                    V   T   V   S   S
14_H                               gtc act gtc tcc tca g  Homsap IGHJ4*02 F
                                    V   T   V   S   S
31_H                               gtc act gtc tcc tca g  Homsap IGHJ4*02 F
                                    V   T   V   S   S
34_H                               gtc act gtc tcc tca g  Homsap IGHJ4*02 F
                                    V   T   V   S   S
42_H                               gtc act gtc tcc tca g  Homsap IGHJ4*02 F
                                    V   T   V   S   S
82_H                               gtc act gtc tcc tca g  Homsap IGHJ4*02 F
```

Sequences aligned with Homsap_IGKV1-39*01

3. V-REGION translation

```
                                   <--------------------------------------------  FR1 - IMGT
                                   1               5                   10                  15
                                    D   I   Q   M   T   Q   S   P   S   S   L   S   A   S   V
X59315 Homsap IGKV1-39*01 F        gac atc cag atg acc cag tct cca tcc tcc ctg tct gca tct gta
                                            V
P3_K                               --- --- gt- --- --- --- --- --- --- --- --- --- --- --- ---
                                            V           R
7_K                                --- --- gt- --- --- -g- --- --- --- --- --- --- --- --- ---
                                            V           R
18_K                               --- --- gt- --- --- -g- --- --- --- --- --- --- --- --- ---
                                            V           R
43_K                               --- --- gt- --- --- -g- --- --- --- --- --- --- --- --- ---
                                            V           R
58_K                               --- --- gt- --- --- -g- --- --- --- --- --- --- --- --- ---
                                            V           R
67_K                               --- --- gt- --- --- -g- --- --- --- --- --- --- --- --- ---
                                            V           R
69_K                               --- --- gt- --- --- -g- --- --- --- --- --- --- --- --- ---
                                            V           R
79_K                               --- --- gt- --- --- -g- --- --- --- --- --- --- --- --- ---
                                            V
61_K                               --- --- gt- --- --- --- --- --- --- --- --- --- --- --- ---
                                            V
74_K                               --- --- gt- --- --- --- --- --- --- --- --- --- --- --- ---
                                            V
5_K                                --- --- gt- --- --- --- --- --- --- --- --- --- --- --- ---
                                            V
14_K                               --- --- gt- --- --- --- --- --- --- --- --- --- --- --- ---
                                            V
31_K                               --- --- gt- --- --- --- --- --- --- --- --- --- --- --- ---
                                            V
34_K                               --- --- gt- --- --- --- --- --- --- --- --- --- --- --- ---
                                            V
```

```
42_K                                --- --- gt- --- --- --- --- --- --- --- --- --- --- --- ---
                                             V
82_K                                --- --- gt- --- --- --- --- --- --- --- --- --- --- --- ---

                                    ------------------------------------------> ________________
                                                    20                  25                  30
                                     G   D   R   V   T   I   T   C   R   A   S   Q   S   I
X59315 Homsap IGKV1-39*01 F         gga gac aga gtc acc atc act tgc cgg gca agt cag agc att ...
                                                                                     T
P3_K                                --- --- --- --- --- --- --- --- --- --- --- --- -c- --- ...
                                                                                     T
7_K                                 --- --- --- --- --- --- --- --- --- --- --- --- -c- --- ...
                                                                                     T
18_K                                --- --- --- --- --- --- --- --- --- --- --- --- -c- --- ...
                                                                                     T
43_K                                --- --- --- --- --- --- --- --- --- --- --- --- -c- --- ...
                                                                                     T
58_K                                --- --- --- --- --- --- --- --- --- --- --- --- -c- --- ...
                                                                                     T
67_K                                --- --- --- --- --- --- --- --- --- --- --- --- -c- --- ...
                                                                                     T
69_K                                --- --- --- --- --- --- --- --- --- --- --- --- -c- --- ...
                                                                                     T
79_K                                --- --- --- --- --- --- --- --- --- --- --- --- -c- --- ...
                                                                                     T
61_K                                --- --- --- --- --- --- --- --- --- --- --- --- -c- --- ...
                                                                                     T
74_K                                --- --- --- --- --- --- --- --- --- --- --- --- -c- --- ...
                                                                                     T
5_K                                 --- --- --- --- --- --- --- --- --- --- --- --- -c- --- ...
                                                                                     T
14_K                                --- --- --- --- --- --- --- --- --- --- --- --- -c- --- ...
                                                                                     T
31_K                                --- --- --- --- --- --- --- --- --- --- --- --- -c- --- ...
                                                                                     T
34_K                                --- --- --- --- --- --- --- --- --- --- --- --- -c- --- ...
                                                                                     T
42_K                                --- --- --- --- --- --- --- --- --- --- --- --- -c- --- ...
                                                                                     T
82_K                                --- --- --- --- --- --- --- --- --- --- --- --- -c- --- ...

                                    __  CDR1 - IMGT  ______________ <---------------------------
                                                    35                  40                  45
                                                         S   S   Y   L   N   W   Y   Q   Q   K
X59315 Homsap IGKV1-39*01 F         ... ... ... ... ... agc agc tat tta aat tgg tat cag cag aaa
                                                                 S       A       F           R
P3_K                                ... ... ... ... ... --- --- -c- --- gc- --- -t- --- --- -g-
                                                                 S       A       F           R
7_K                                 ... ... ... ... ... --- --- -c- --- gc- --- -t- --- --- -g-
                                                                 S       A       F           R
18_K                                ... ... ... ... ... --- --- -c- --- gc- --- -t- --- --- -g-
                                                                 S       A       F           R
43_K                                ... ... ... ... ... --- --- -c- --- gc- --- -t- --- --- -g-
                                                                 S       A       F           R
58_K                                ... ... ... ... ... --- --- -c- --- gc- --- -t- --- --- -g-
                                                                 S       A       F           R
67_K                                ... ... ... ... ... --- --- -c- --- gc- --- -t- --- --- -g-
                                                                 S       A       F           R
69_K                                ... ... ... ... ... --- --- -c- --- gc- --- -t- --- --- -g-
                                                                 S       A       F           R
79_K                                ... ... ... ... ... --- --- -c- --- gc- --- -t- --- --- -g-
                                                                 S       A       F           R
61_K                                ... ... ... ... ... --- --- -c- --- gc- --- -t- --- --- -g-
                                                                 S       A       F           R
74_K                                ... ... ... ... ... --- --- -c- --- gc- --- -t- --- --- -g-
                                                                 S       A       F           R
5_K                                 ... ... ... ... ... --- --- -c- --- gc- --- -t- --- --- -g-
                                                                 S       A       F           R
14_K                                ... ... ... ... ... --- --- -c- --- gc- --- -t- --- --- -g-
                                                                 S       A       F           R
31_K                                ... ... ... ... ... --- --- -c- --- gc- --- -t- --- --- -g-
                                                                 S       A       F           R
34_K                                ... ... ... ... ... --- --- -c- --- gc- --- -t- --- --- -g-
                                                                 S       A       F           R
42_K                                ... ... ... ... ... --- --- -c- --- gc- --- -t- --- --- -g-
```

```
                                                                 S       A       F           R
82_K                                ... ... ... ... ... --- --- -c- --- gc- --- -t- --- --- -g-

                                     FR2 - IMGT  --------------------------> ______________  CDR2
                                                    50                  55                  60
                                     P   G   K   A   P   K   L   L   I   Y   A   A
X59315 Homsap IGKV1-39*01 F         cca ggg aaa gcc cct aag ctc ctg atc tat gct gca ... ... ...
                                             E           N                   S
P3_K                                --g --a g-- --- --- --c --- --- --- --- ag- --- ... ... ...
                                             E           N                   S
7_K                                 --g --a g-- --- --- --c --- --- --- --- ag- --- ... ... ...
                                             E           N                   S
18_K                                --g --a g-- --- --- --c --- --- --- --- ag- --- ... ... ...
                                             E           N                   S
43_K                                --g --a g-- --- --- --c --- --- --- --- ag- --- ... ... ...
                                             E           N                   S
58_K                                --g --a g-- --- --- --c --- --- --- --- ag- --- ... ... ...
                                             E           N                   S
67_K                                --g --a g-- --- --- --c --- --- --- --- ag- --- ... ... ...
                                             E           N                   S
69_K                                --g --a g-- --- --- --c --- --- --- --- ag- --- ... ... ...
                                             E           N                   S
79_K                                --g --a g-- --- --- --c --- --- --- --- ag- --- ... ... ...
                                             E           N                   S
61_K                                --g --a g-- --- --- --c --- --- --- --- ag- --- ... ... ...
                                             E           N                   S
74_K                                --g --a g-- --- --- --c --- --- --- --- ag- --- ... ... ...
                                             E           N                   S
5_K                                 --g --a g-- --- --- --c --- --- --- --- ag- --- ... ... ...
                                             E           N                   S
14_K                                --g --a g-- --- --- --c --- --- --- --- ag- --- ... ... ...
                                             E           N                   S
31_K                                --g --a g-- --- --- --c --- --- --- --- ag- --- ... ... ...
                                             E           N                   S
34_K                                --g --a g-- --- --- --c --- --- --- --- ag- --- ... ... ...
                                             E           N                   S
42_K                                --g --a g-- --- --- --c --- --- --- --- ag- --- ... ... ...
                                             E           N                   S
82_K                                --g --a g-- --- --- --c --- --- --- --- ag- --- ... ... ...

                                     - IMGT  __________ <----------------------------------------
                                                    65                  70                  75
                                                     S   S   L   Q   S   G   V   P       S   R
X59315 Homsap IGKV1-39*01 F         ... ... ... ... tcc agt ttg caa agt ggg gtc cca ... tca agg

P3_K                                ... ... ... ... --- --- --- --- --- --- --- --- ... --- ---
                                                         N
7_K                                 ... ... ... ... --- -a- --- --- --- --- --- --- ... --- ---
                                                         N
18_K                                ... ... ... ... --- -a- --- --- --- --- --- --- ... --- ---
                                                         N
43_K                                ... ... ... ... --- -a- --- --- --- --- --- --- ... --- ---
                                                         N
58_K                                ... ... ... ... --- -a- --- --- --- --- --- --- ... --- ---
                                                         N
67_K                                ... ... ... ... --- -a- --- --- --- --- --- --- ... --- ---
                                                         N
69_K                                ... ... ... ... --- -a- --- --- --- --- --- --- ... --- ---
                                                         N
79_K                                ... ... ... ... --- -a- --- --- --- --- --- --- ... --- ---

61_K                                ... ... ... ... --- --- --- --- --- --- --- --- ... --- ---

74_K                                ... ... ... ... --- --- --- --- --- --- --- --- ... --- ---

5_K                                 ... ... ... ... --- --- --- --- --- --- --- --- ... --- ---

14_K                                ... ... ... ... --- --- --- --- --- --- --- --- ... --- ---

31_K                                ... ... ... ... --- --- --- --- --- --- --- --- ... --- ---

34_K                                ... ... ... ... --- --- --- --- --- --- --- --- ... --- ---

42_K                                ... ... ... ... --- --- --- --- --- --- --- --- ... --- ---
```

```
82_K                                ... ... ... ... --- --- --- --- --- --- --- --- ... --- ---

                                    ------------------------------- FR3 - IMGT ---------------
                                                    80                  85                  90
                                     F   S   G   S   G           S   G   T   D   F   T   L   T
X59315 Homsap IGKV1-39*01 F         ttc agt ggc agt gga ... ... tct ggg aca gat ttc act ctc acc

P3_K                                --- --- --- --- --- ... ... --- --- --- --- --- --- --- ---

7_K                                 --- --- --- --- --- ... ... --- --- --- --- --- --- --- ---

18_K                                --- --- --- --- --- ... ... --- --- --- --- --- --- --- ---

43_K                                --- --- --- --- --- ... ... --- --- --- --- --- --- --- ---

58_K                                --- --- --- --- --- ... ... --- --- --- --- --- --- --- ---

67_K                                --- --- --- --- --- ... ... --- --- --- --- --- --- --- ---

69_K                                --- --- --- --- --- ... ... --- --- --- --- --- --- --- ---

79_K                                --- --- --- --- --- ... ... --- --- --- --- --- --- --- ---

61_K                                --- --- --- --- --- ... ... --- --- --- --- --t --- --- ---

74_K                                --- --- --- --- --- ... ... --- --- --- --- --t --- --- ---

5_K                                 --- --- --t --- --- ... ... --- --- --- --- --- --c --- ---

14_K                                --- --- --t --- --- ... ... --- --- --- --- --- --c --- ---

31_K                                --- --- --t --- --- ... ... --- --- --- --- --- --c --- ---

34_K                                --- --- --t --- --- ... ... --- --- --- --- --- --c --- ---

42_K                                --- --- --t --- --- ... ... --- --- --- --- --- --c --- ---

82_K                                --- --- --t --- --- ... ... --- --- --- --- --- --c --- ---

                                    ----------------------------------------------------> ____
                                                    95                 100             104
                                     I   S   S   L   Q   P   E   D   F   A   T   Y   Y   C   Q
X59315 Homsap IGKV1-39*01 F         atc agc agt ctg caa cct gaa gat ttt gca act tac tac tgt caa
                                                                                     S
P3_K                                --- --- --- --t --- --- --- --- --- --- --- --- -c- --- ---
                                             G                                       S
7_K                                 --- --- g-- --t --- --- --- --- --- --- --- --- -c- --- ---
                                             G                                       S
18_K                                --- --- g-- --t --- --- --- --- --- --- --- --- -c- --- ---
                                             G                                       S
43_K                                --- --- g-- --t --- --- --- --- --- --- --- --- -c- --- ---
                                             G                                       S
58_K                                --- --- g-- --t --- --- --- --- --- --- --- --- -c- --- ---
                                             G                                       S
67_K                                --- --- g-- --t --- --- --- --- --- --- --- --- -c- --- ---
                                             G                                       S
69_K                                --- --- g-- --t --- --- --- --- --- --- --- --- -c- --- ---
                                             G                                       S
79_K                                --- --- g-- --t --- --- --- --- --- --- --- --- -c- --- ---
                                                                                     S
61_K                                --- --- --- --t --- --- --- --- --- --- --- --- -c- --- ---
                                                                                     S
74_K                                --- --- --- --t --- --- --- --- --- --- --- --- -c- --- ---
                                                                                     S
5_K                                 --- --- --- --t --- --- --- --- --- --- --- --- -c- --- ---
                                                                                     S
14_K                                --- --- --- --t --- --- --- --- --- --- --- --- -c- --- ---
                                                                                     S
31_K                                --- --- --- --t --- --- --- --- --- --- --- --- -c- --- ---
                                                                                     S
34_K                                --- --- --- --t --- --- --- --- --- --- --- --- -c- --- ---
                                                                                     S
42_K                                --- --- --- --t --- --- --- --- --- --- --- --- -c- --- ---
                                                                                     S
82_K                                --- --- --- --t --- --- --- --- --- --- --- --- -c- --- ---
```

```
                                    __  CDR3 - IMGT  __

                                     Q   S   Y   S   T   P
X59315 Homsap IGKV1-39*01 F         cag agt tac agt acc cct cc
                                         T           A       P   T   F   G   G   G   T   K   L
P3_K                                --- -c- --- --- g-- --- --c act ttc ggc gga ggg acc aag ctg
                                         T           A       P   T   F   G   G   G   T   K   V
7_K                                 --- -c- --- --- g-- --- --c act ttc ggc gga ggg acc aag gtg
                                         T           A       P   T   F   G   G   G   T   K   V
18_K                                --- -c- --- --- g-- --- --c act ttc ggc gga ggg acc aag gtg
                                         T           A       P   T   F   G   G   G   T   K   V
43_K                                --- -c- --- --- g-- --- --c act ttc ggc gga ggg acc aag gtg
                                         T           A       P   T   F   G   G   G   T   K   V
58_K                                --- -c- --- --- g-- --- --c act ttc ggc gga ggg acc aag gtg
                                         T           A       P   T   F   G   G   G   T   K   V
67_K                                --- -c- --- --- g-- --- --c act ttc ggc gga ggg acc aag gtg
                                         T           A       P   T   F   G   G   G   T   K   V
69_K                                --- -c- --- --- g-- --- --c act ttc ggc gga ggg acc aag gtg
                                         T           A       P   T   F   G   G   G   T   K   V
79_K                                --- -c- --- --- g-- --- --c act ttc ggc gga ggg acc aag gtg
                                         T           A       P   T   F   G   G   G   T   K   V
61_K                                --- -c- --- --- g-- --- --c act ttc ggc gga ggg acc aaa gtg
                                         T           A       P   T   F   G   G   G   T   K   V
74_K                                --- -c- --- --- g-- --- --c act ttc ggc gga ggg acc aaa gtg
                                         T           A       P   T   F   G   G   G   T   K   L
5_K                                 --- -c- --- --- g-- --- --c act ttc ggc gga ggg acc aag ctg
                                         T           A       P   T   F   G   G   G   T   K   L
14_K                                --- -c- --- --- g-- --- --c act ttc ggc gga ggg acc aag ctg
                                         T           A       P   T   F   G   G   G   T   K   L
31_K                                --- -c- --- --- g-- --- --c act ttc ggc gga ggg acc aag ctg
                                         T           A       P   T   F   G   G   G   T   K   L
34_K                                --- -c- --- --- g-- --- --c act ttc ggc gga ggg acc aag ctg
                                         T           A       P   T   F   G   G   G   T   K   L
42_K                                --- -c- --- --- g-- --- --c act ttc ggc gga ggg acc aag ctg
                                         T           A       P   T   F   G   G   G   T   K   L
82_K                                --- -c- --- --- g-- --- --c act ttc ggc gga ggg acc aag ctg



X59315 Homsap IGKV1-39*01 F

                                     E   I   K
P3_K                                gag atc aaa g  Homsap IGKJ4*01 F
                                     E   I   K
7_K                                 gag atc aaa g  Homsap IGKJ4*01 F
                                     E   I   K
18_K                                gag atc aaa g  Homsap IGKJ4*01 F
                                     E   I   K
43_K                                gag atc aaa g  Homsap IGKJ4*01 F
                                     E   I   K
58_K                                gag atc aaa g  Homsap IGKJ4*01 F
                                     E   I   K
67_K                                gag atc aaa g  Homsap IGKJ4*01 F
                                     E   I   K
69_K                                gag atc aaa g  Homsap IGKJ4*01 F
                                     E   I   K
79_K                                gag atc aaa g  Homsap IGKJ4*01 F
                                     D   I   K
61_K                                gat atc aaa g  Homsap IGKJ4*01 F
                                     D   I   K
74_K                                gat atc aaa g  Homsap IGKJ4*01 F
                                     E   I   K
5_K                                 gag atc aaa g  Homsap IGKJ4*01 F
                                     E   I   K
14_K                                gag atc aaa g  Homsap IGKJ4*01 F
                                     E   I   K
31_K                                gag atc aaa g  Homsap IGKJ4*01 F
                                     E   I   K
34_K                                gag atc aaa g  Homsap IGKJ4*01 F
                                     E   I   K
42_K                                gag atc aaa g  Homsap IGKJ4*01 F
                                     E   I   K
82_K                                gag atc aaa g  Homsap IGKJ4*01 F
```
