# Supplementary material for: Pacific Biosciences Sequencing and IMGT/HighV-QUEST Analysis of Full-Length Single Chain Fragment Variable from an In Vivo Selected Phage-Display Combinatorial Library
Source: Front Immunol. 2017 Dec 20;8:1796. doi: 10.3389/fimmu.2017.01796 (PMC5742356; doi:10.3389/fimmu.2017.01796)
Supplement: Supplementary file 6 [file table_1.docx]

Supplementary Material

**NGS PacBio full-length sequencing and IMGT/HighV-QUEST analysis of scFv from a phage-display combinatorial library**

Audrey Hemadou, Véronique Giudicelli, Melissa Smith, Marie-Paule Lefranc*, Patrice Duroux, Sofia Kossida, Cheryl Heiner, John Kuijpers, Alexis Groppi, Jonas Korlach, Philippe Mondon, Florence Ottones, Marie-Josée Jacobin-Valat, Jeanny Laroche-Traineau and Gisèle Clofent-Sanchez*

*** Correspondence:**Marie-Paule Lefranc [Marie-Paule.Lefranc@igh.cnrs.fr](mailto:Marie-Paule.Lefranc@igh.cnrs.fr)
Gisèle Clofent-Sanchez [gisele.clofent-sanchez@rmsb.u-bordeaux2.fr](mailto:gisele.clofent-sanchez@rmsb.u-bordeaux2.fr)

# Supplementary Tables

# Table S1. Repartition of the AAR3 scFv, in the 15 SMRT Cells, based on their format (VH-VL or VL-VH, VH-VH, VL-VL) determined by IMGT/HighV-QUEST using the novel functionality for scFv.

| PCR  sample N° | Number of reads with 99.9% predicted accuracy | SMRT Cell  N° | Number of PacBio  CCS2 analysed reads | Number of filtered-in reads | Number of VH-VL or VL-VH | Number of VH-VH | Number of VL-VL |
| --- | --- | --- | --- | --- | --- | --- | --- |
| s1 | 91828 | 1 | 29,224 | 22,906 | 22,819 | 13 | 74 |
|  |  | 2 | 32,240 | 25,228 | 25,136 | 14 | 78 |
|  |  | 3 | 30,364 | 23,799 | 23,723 | 8 | 68 |
| s2 | 129640 | 4 | 34,082 | 26,657 | 26,518 | 22 | 117 |
|  |  | 5 | 33,510 | 26,407 | 26,284 | 11 | 112 |
|  |  | 6 | 31,980 | 25,032 | 24,903 | 5 | 124 |
|  |  | 7 | 30,068 | 23,695 | 23,605 | 13 | 77 |
| s3 | 115446 | 8 | 34,890 | 26,990 | 26,817 | 15 | 158 |
|  |  | 9 | 29,373 | 22,468 | 22,343 | 7 | 118 |
|  |  | 10 | 26,465 | 20,044 | 19,959 | 6 | 79 |
|  |  | 11 | 24,718 | 18,741 | 18,597 | 12 | 132 |
| s4 | 113644 | 12 | 25,128 | 19,120 | 19,027 | 9 | 84 |
|  |  | 13 | 23,693 | 17,756 | 17,670 | 8 | 78 |
|  |  | 14 | 32,293 | 24,762 | 24,634 | 10 | 118 |
|  |  | 15 | 32,530 | 25,054 | 24,899 | 18 | 137 |
| Total |  |  | 450,558 | 348,659 | 34,6934 | 171 | 1,554 |

**Figure S1.** PacBio general workflow for amplicon sample preparation^4^ (**A**) and SMRTbell librairies loading and sequencing (**B**).

(<http://www.pacb.com/wp-content/uploads/2014/04/TemplatePreparation.pdf>)

**Figure S2**. Scheme of the four PCR amplifications of the AAR3 fraction and design of the corresponding 15 SMRT Cells. Sample 1 was loaded on 3 SMRT cells, and not 4, owing to limited sample quantity after library preparation.

**Figure S3**. Characterisation of P3 by IMGT/V-QUEST. (A) Parameters and positions of the two V domains VH and VL and of the linker. (B) Identification of the IMGT clonotype (AA) for VH domain (IGHV6-1*01 - IGHD6-13*01 - IGHJ4*02 [10.9.10]; CARQGSTYFDYW). The domain is unproductive because of a stop codon in FR2-IMGT position 52 according to the IMGT unique numbering (Lefranc et al. Dev. Comp. Immunol., 27, 55-77 (2003)). For biological analysis the stop codon was replaced with the tgg codon of the IGHV6-1*01. (C) Identification of the IMGT clonotype (AA) for VK domain: (IGKV1-39*01 - IGKJ4*01 [6.3.9]; CQQTYSAPPTF).

**Figure S4.** Alignment of the 25 P3-related reads with one of the P3 reads (among the fully identical on 977 bp). Dashes indicate alignment gaps. Numbers on the right indicate the number of nucleotides in the P3 sequence (excluding gaps created by the alignment). Primer FWD, VH, Linker, VL, Primer REV are delimited above the sequence. Mutations by comparison to P3 are highlighted in red, excepted those in the primers (only deletions) which are shown in pink. Large deletions at the 3’ end of P3-related 58 and 81 are left in blank.

**Figure S5.** Alignment of the 15 P3-related reads originating from the scFv phagemid combinatorial library with the P3 Sanger sequence. The alignment was performed using IMGT/V-QUEST software (program version: 3.4.8 - reference directory release: [201746-2](http://www.imgt.org/IMGT_vquest/share/textes/datareleases.html)).
